# Origin of the plant *Tm-1-like* gene *via* two independent horizontal transfer events and one gene fusion event

**DOI:** 10.1038/srep33691

**Published:** 2016-09-20

**Authors:** Zefeng Yang, Li Liu, Huimin Fang, Pengcheng Li, Shuhui Xu, Wei Cao, Chenwu Xu, Jinling Huang, Yong Zhou

**Affiliations:** 1 Jiangsu Key Laboratory of Crop Genetics and Physiology/Co-Innovation Center for Modern Production Technology of Grain Crops, Key Laboratory of Plant Functional Genomics of the Ministry of Education, Yangzhou University, Yangzhou, 225009, China; 2 Department of Biology, East Carolina University, Greenville, NC, 27858, USA

## Abstract

The *Tomato mosaic virus* (ToMV) resistance gene *Tm-1* encodes a direct inhibitor of ToMV RNA replication to protect tomato from infection. The plant Tm-1-like (Tm-1L) protein is predicted to contain an uncharacterized N-terminal UPF0261 domain and a C-terminal TIM-barrel signal transduction (TBST) domain. Homologous searches revealed that proteins containing both of these two domains are mainly present in charophyte green algae and land plants but absent from glaucophytes, red algae and chlorophyte green algae. Although Tm-1 homologs are widely present in bacteria, archaea and fungi, UPF0261- and TBST-domain-containing proteins are generally encoded by different genes in these linages. A co-evolution analysis also suggested a putative interaction between UPF0261- and TBST-domain-containing proteins. Phylogenetic analyses based on homologs of these two domains revealed that plants have acquired UPF0261- and TBST-domain-encoding genes through two independent horizontal gene transfer (HGT) events before the origin of land plants from charophytes. Subsequently, gene fusion occurred between these two horizontally acquired genes and resulted in the origin of the *Tm-1L* gene in streptophytes. Our results demonstrate a novel evolutionary mechanism through which the recipient organism may acquire genes with functional interaction through two different HGT events and further fuse them into one functional gene.

Green plants, also known as Viridiplantae, consist of green algae (Chlorophyta and Streptophyta) and land plants (embryophytes). The difference between Streptophyta and Chlorophyta is correlated with a remarkably conservative preference for freshwater/marine habitats, and the early freshwater adaptation of streptophyte algae was a major advantage for the colonization of land by plants^1^. The origin of land plants is one of the most important events in the evolution of life on Earth and is the key step in the process of developing of modern terrestrial ecosystems. From peaceably aquatic surroundings to harshly terrestrial environments, the ancestor of land plants had to face significant stresses, including dryness, ultraviolet radiation and pathogenic microorganisms other than those in water. During their colonization of land, plants gradually evolved new genes and phenotypic novelties to adapt to and radiate in terrestrial environments^2^,^3^. The genome of *Klebsormidium flaccidum* reveals that this filamentous terrestrial alga has acquired many genes, including those producing several plant hormones, and a primitive system to protect against the harmful effects of high-intensity light^4^. Because plants are constantly exposed to microbes, the evolution of land plants has been shaped by molecular interactions with epiphytic, symbiotic and pathogenic microbes. Thus, evolution of new genes for resistance to pathogenic microorganisms was extremely important for plants to colonize the land^5^.

The tomato (*Solanum lycopersicum* L.) gene *Tm-1*, conferring resistance to *Tomato mosaic virus* (ToMV), was bred from the wild tomato *S. habrochaites*^6^,^7^. Although the Tm-1 protein does not share any functional domain with previously known resistance (R) proteins, it physically binds ToMV replication proteins and functionally inhibits the RNA-dependent RNA replication of ToMV^8^,^9^. In *S. habrochaites*, a small region of *Tm-1* was under positive selection during its antagonistic coevolution with ToMV, and the positively selected residues have evolved to counter ToMV infection and are important for the inhibition of ToMV RNA replication^10^. Further evidence also confirmed this coevolution relationship between *Tm-1* and ToMV, because a single naturally occurring amino acid change in the positively selected region of Tm-1 enables it to inhibit the replication of a *Tm-1* resistance-breaking ToMV mutant^6^,^10^. The tomato Tm-1 protein is predicted to contain two evolutionarily conserved domains: an uncharacterized N-terminal domain and a C-terminal TIM-barrel-like domain^8^. Experimental evidence has revealed that the Tm-1 N-terminal region was responsible for its inhibitory activity, and a C-terminally truncated Tm-1 protein still possesses inhibitory activity^6^,^7^. These findings also suggest that the full-length tomato Tm-1 has a function other than ToMV resistance^11^.

The homologs of the tomato *Tm-1* gene are present in a wide range of land plants^11^. Although whether these *Tm-1* homologs help to inhibit virus multiplication is unknown, the ubiquity of this gene in land plants suggests that its functions include a wide range of selectivity. In this work, we tried to obtain more insights into the origin of the plant *Tm-1-like* (*Tm-1L*) gene by performing an extensive search of its homologs in current sequence databases and by analyzing their phylogeny.

## Results

### The *Tm-1L* genes are widespread in streptophytes

**Figure 1:**
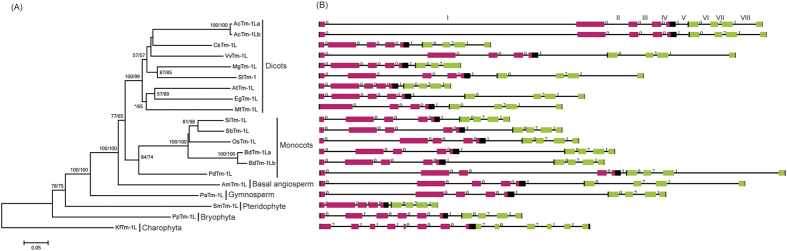
The phylogenetic tree of the green plant *Tm-1L* genes (**A**) and their exon/intron structures (**B**). The numbers above the branches represent the bootstrap values for the maximum likelihood and distance analyses, respectively. The asterisks indicate values <50%. The exons are indicated by boxes, whereas introns are indicated by lines. The UPF0261 domain regions are indicated by red boxes, while the TBST domain regions are indicated by green boxes. The number above an intron indicates the phase.

BLAST searches revealed that homologs of the tomato *Tm-1* gene are present in various streptophyte groups, including charophyte algae, bryophytes, lycophytes and seed plants. In addition, we also noticed that nearly all of the sequenced land plant genomes contained at least one *Tm-1L* gene. However, no homologs were detected in the genomes of chlorophyte algae. To investigate the origin and evolution of the land plant *Tm-1L* genes, we characterized them from species representing the main lineages of streptophytes, including the charophyte alga *K. flaccidum*, the bryophtye *Physcomitrella patens*, the lycophyte *Selaginella moellendorffii*, the gymnosperm *Picea abies*, the basal angiosperm *Amborella trichopoda* and five monocot and eight dicot angiosperms (Supplementary Table S1). Among the tested plant genomes, two (dicot *Aquilegia coerulea* and monocot *Brachypodium distachyon*) contained two *Tm-1L* genes, while all of the other genomes contained only one. In the phylogeny (Fig. 1), both of these paralogous genes were located at the termini of branches, indicating that these paralogs were formed through recent duplication events. Further investigation revealed that both paralogous pairs were results of segmental duplication because there were highly conserved genes within the flanking regions of these two pairs of paralogous *Tm-1L* genes.

Most of the land plant *Tm-1L* genes generally contained eight introns in their coding regions, suggesting a highly similar gene structure among them, and the positions and phases of these eight introns were conserved. These results also suggest that the main characteristics of the gene structure of this family were formed in the common ancestor of land plants. Some intron gain/loss events were also found in the evolution of land plant *Tm-1L* genes. For example, the *P. patens PpTm-1L* gene contained nine introns in the coding region, and an extra intron was inserted in the second exon. In addition, the *Medicago truncatula MtTm-1L* and *Mimulus guttatus MgTm-1L* genes contained only seven introns in the coding regions, and a gene structure comparison suggested that the *MtTm-1L* gene had lost the first intron, while the *MgTm-1L* gene had lost the last one. However, the *Kf Tm-1L* gene in the charophyte alga *K*. *f laccidum* possessed 11 introns in its coding regions, and the positions and phases of these introns were significantly different from those in land plants.

### The phylogenetic relationship of the *Tm-1L* homologs

To investigate the origin of the streptophyte *Tm-1L* genes, we searched the *nr* and EST databases of NCBI and the available eukaryotic genome databases for Tm-1L homologs. However, no homologs in any other algae were detected, including glaucophytes, red algae and chlorophyte green algae. This result suggests that the plant *Tm-1L* gene might have first appeared in streptophyte algae. Blast results also reveal that homologs of plant Tm-1L proteins only exist in prokaryotes, fungi and *Acanthamoeba castellanii*, a species of Amoebozoa. To our surprise, only two genes in *Aspergillus*, a fungi genus, showed global similarity with plant *Tm-1L* genes, while the genes in prokaryotes and other fungi only possessed partial homeologous sequences. Some prokaryotic and fungal proteins only possess a UPF0261 domain and show high similarity with the N terminus of plant Tm-1L proteins. However, other prokaryotic and fungal proteins only possess a TBST domain and show high similarity with the C terminus of plant Tm-1L proteins. It is difficult to build a phylogenetic tree using the whole sequences of Tm-1L homologs due to a lack of consistent sequences. Thus, two phylogenetic trees were constructed using the UPF0261 and TBST domain sequences, respectively.

**Figure 2:**
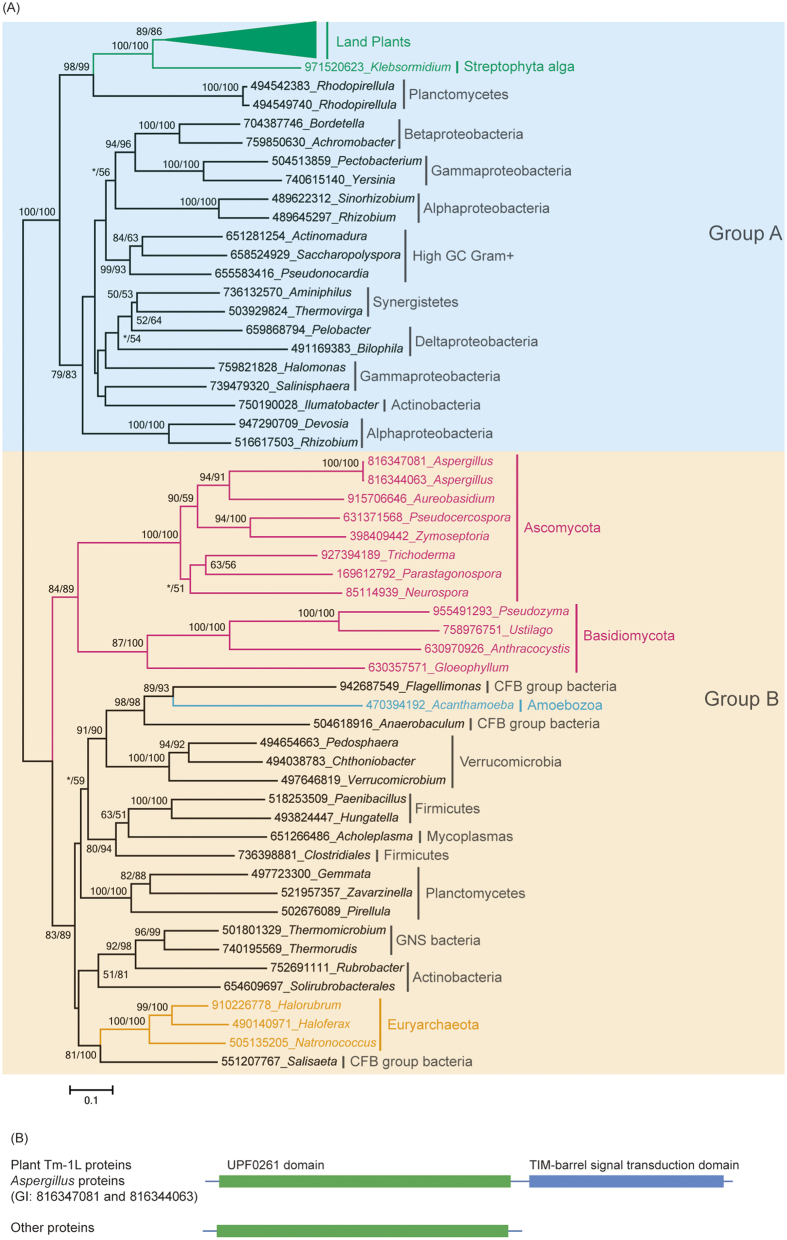
Phylogenetic analyses and the domain structures of the genes encoding UPF0261-domain-containing proteins. **(A)** Phylogenetic analyses of the proteins containing a UPF0261 domain. The numbers above the branches represent the bootstrap values for the maximum likelihood and distance analyses. All of the sequences were obtained from NCBI, except for those in the green plants, and each protein is indicated by the GI numbers in NCBI and its genus. **(B)** The domain structure of the proteins that were used in the phylogeny.

We first selected representative homologs of proteins possessing a UPF0261 domain from each taxonomic group of cellular organisms in the *nr* database to build a phylogenetic tree (Fig. 2). The genes encoding proteins with UPF0261 domains are distributed widely in bacteria, suggesting that this gene first emerged in bacteria. In addition, the molecular phylogeny of the UPF0261 domain consists of two distinct groups with high bootstrap support values. All of the genes in streptophyte plants were in group A, while their homologs in fungi were in group B. Group A only contained genes from bacteria and plants, and all of the plant UPF0261-domain-containing proteins formed a single clade with high bootstrap support. The monophyly of the plant UPF0261-domain-containing proteins strongly suggests that they have a single origin and are derived from a unique gene that was already present in streptophyte algae. The plant UPF0261-domain-containing proteins fell within the branch of planctomycete genes, showing high bootstrap support values in both maximum likelihood and distance analyses. These observations indicate that the origin of plant UPF0261-domain-containing protein genes is a result of horizontal gene transfer and that the putative donor might be a planctomycete.

Group B contained genes from fungi, amoebozoa, bacteria and archaea. The fungal genes encoding a UPF0261-domain-containing protein formed a single clade with high bootstrap support. However, blast searches revealed that fungal UPF0261-domain-containing proteins are only present in the subkingdom Dikarya. The monophyly of the fungal UPF0261-domain-containing protein orthologs strongly indicates that they have a single origin and are derived from a gene that was already present at least in the ancestor of Dikarya. These results also suggest that the origin of this gene in fungi is another HGT event from prokaryotes. The genome of the free-living amoeba species *Acanthamoeba castellanii* also possesses an UPF0261-domain-containing protein gene that is located in group B and falls into the branch of bacteria. There were no homologs in close relatives of *A. castellanii*. This result suggests that the origin of the UPF0261-domain-containing protein gene in *A. castellanii* is another horizontal gene transfer event from bacteria.

**Figure 3:**
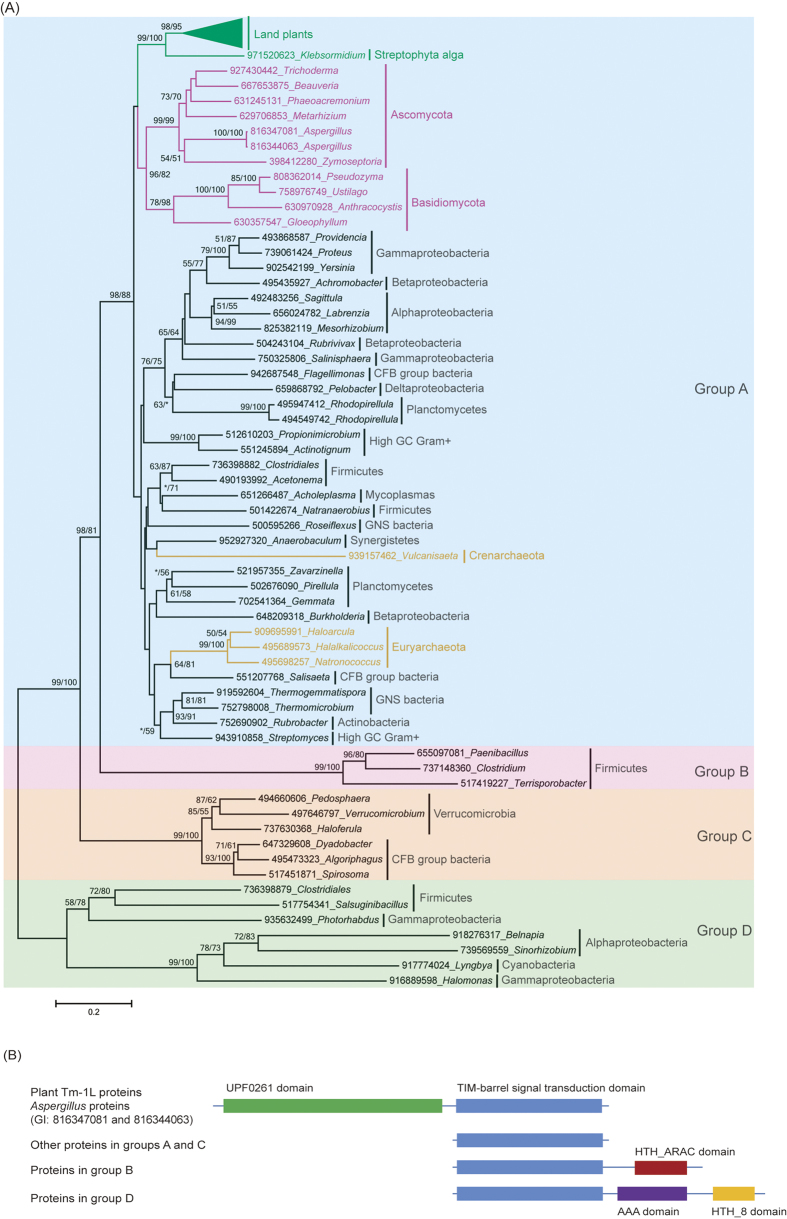
Phylogenetic analyses and the domain structures of the genes encoding TBST-domain-containing proteins. **(A)** Phylogenetic analyses of the proteins containing a TBST domain. The numbers above the branches represent the bootstrap values for the maximum likelihood and distance analyses. All of the sequences were obtained from the NCBI, except for those in the green plants, and each protein is indicated by the GI numbers in NCBI and its genus. **(B)** The domain structure of the proteins that were used in the phylogeny.

**Figure 4:**
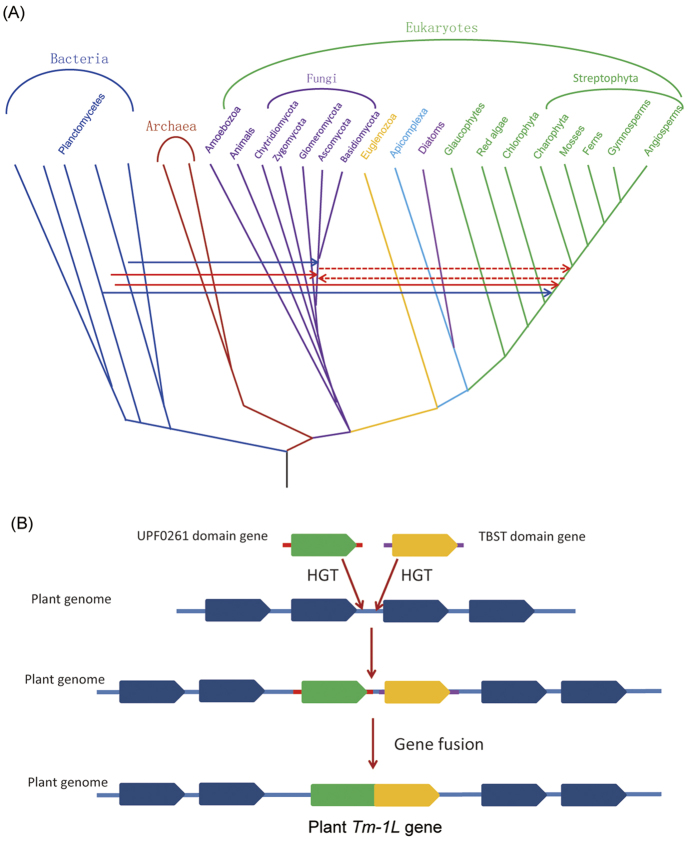
An evolutionary scenario for the origin of the plant *Tm-1L* gene. **(A)** Horizontal gene transfer events associated with the origin of Tm-1 homologs in land plants and fungi. Solid blue arrows indicate the HGT of the UPF0261-domain-containing gene. Two solid red arrows indicate the HGT of the TBST-domain-containing gene from prokaryotes to plants and fungi, respectively. Dashed red arrows indicate the HGT of the TBST-domain-containing gene between plants and fungi. (**B**) Evolutionary history of the green plant *Tm-1L* gene.

Second, we also selected representative homologs of proteins possessing a TBST domain from each taxonomic group of cellular organisms in the *nr* database to build a phylogenetic tree (Fig. 3). According to the phylogenetic tree and the domain structure, the TBST-domain-containing proteins were divided into four evolutionarily distinct groups. Group A contained genes from bacteria, archaea and eukaryotes, while the other groups only possessed genes from bacteria. The protein sequences within each group showed high similarity, while those between groups were quite divergent. On the phylogenetic tree of TBST-domain-containing proteins, the eukaryotic cluster exclusively contained orthologs from green plants and fungi but no other eukaryotic lineage, forming two well-supported monophyletic sister groups. The genes encoding TBST-domain-containing proteins were widely distributed in prokaryotes and mainly in bacteria. The universality of their distribution in bacteria also suggests that this gene first emerged in bacteria. Both the plant and fungal TBST-domain-containing proteins formed a single clade with high bootstrap support, suggesting that they have a single origin in these two lineages, respectively. The evolutionary relationship of TBST homologs from plants and fungi clearly indicates a common origin. However, the kingdom Plantae does not share a specific ancestor with fungi except for the common ancestor of eukaryotes^12^,^13^. The first scenario for the origin of the *TBST* gene in eukaryotes is that it was present in the ancestor of all eukaryotes. However, this scenario requires too many independent gene loss events, which seems unlikely. The second scenario is that a *TBST* gene originated either in the ancestor of green plants or in the ancestor of at least Dikarya fungi through one HGT event and then was transferred *via* another HGT event between these two subkingdoms (Fig. 4A). The third scenario is that two independent HGT events transferred the *TBST* gene from prokaryotes to the ancestor of land plants and to the ancestor of Dikarya fungi, respectively. Although we cannot confirm which of the last two scenarios is more parsimonious, at least one HGT event is suggested to contribute to the origin of the TBST domain in streptophyte plant *Tm-1L* genes.

### Co-evolution between the genes encoding UPF0261 and TBST proteins

To evaluate the possible interaction between the proteins containing UPF0261 and TBST domains, the Mirrortree server^14^ was used to access the co-evolution between these two protein families in bacteria, archaea and fungi. When we used the sequences of the UPF0261 and TBST domain regions of the *Arabidopsis* Tm-1L protein as the reference sequences, a total of 159 pairs of homologs were selected by the Mirrortree server. An analysis of the full-length UPF0261 and TBST sequences from bacteria, archaea and fungi showed that the phylogenies of these two protein families are topologically similar (Supplementary Figure S1). Mirrortree analysis revealed that the correlation coefficient of these trees was 0.886 (*P* < 0.0001) (Table 1), suggesting the co-evolution of both proteins across bacteria, archaea and fungi.

In the NCBI RefSeq protein database, at least 714 TBST-domain-containing proteins were carried by 504 bacterial genomes. Among these genomes, 491 (97.4%) contained genes encoding UPF0261 proteins. A total of 338 genomes contained only one TBST-domain-encoding gene and only one UPF0261-domain-encoding gene. We selected the 333 genomes to evaluate the co-evolution between TBST and UPF0261 genes in bacteria because the other five genomes carried genes with truncated TBST and/or UPF0261 domains (Supplementary Table S2). The pairwise distances between sequences of the *TBST* genes were strongly correlated with those between *UPF0261* genes (Table 1), and the estimated correlation coefficient *r* was 0.7735 (*P* < 0.0001). Furthermore, a correlation between the TBST and UPF0261 genes was also found in archaea and fungi. In the NCBI RefSeq database, 24 archaea genomes were found to contain *TBST* genes, and a total of 22 genomes (91.67%) had only one TBST gene and only one *UPF0261* gene. The estimated *r* was 0.7185, which was also significantly higher than zero at the level of *P* < 0.0001. In fungi, 68 genomes contained *TBST* genes, and 61 (89.7%) carried only one *TBST* gene and one *UPF0261* gene. The estimated *r* was 0.8067. When we used all of these bacteria, archaea and fungi genomes to perform a correlation analysis for the distance matrices between TBST and UPF0261 proteins, the estimated *r* was 0.7192. These results indicate that the TBST and UPF0261 proteins underwent highly correlated co-evolution in bacteria, archaea and fungi. In addition, the TBST and UPF0261 domains in plant Tm-1 proteins were also used to investigate their possible domain-domain coevolution. The estimated *r* between the distances of the two domains was 0.8675 (*P* < 0.0001), suggesting that highly correlated co-evolution between the TBST and UPF0261 domains in Tm-1 proteins occurred during plant evolution. However, the correlation coefficient in plants was not statistically larger than the average value in bacteria, archaea and fungi (*P* = 0.0778, one-sample *t*-test).

## Discussion

### Horizontal gene transfer and the origin of plant *Tm-1L* genes

Horizontal gene transfer (HGT), also known as lateral gene transfer (LGT), refers to the transformation of genetic material between organisms with reproductive isolation^15^. HGT has been thought to be one of the most important evolutionary forces and to be frequent only within prokaryotes and certain unicellular eukaryotes^16^,^17^. Recent genome analyses also detected horizontally acquired genes in all major lineages of multicellular eukaryotes, including plants, fungi and animals^18^,^19^,^20^,^21^, suggesting that HGT was also critical for adaptive evolution throughout eukaryotic evolution. In green plants, many horizontally acquired genes were also found to be of adaptive and evolutionary importance^22^. Because intimate physical association may facilitate HGT, parasitic plants have been used to detect HGT^23^,^24^. For instance, based on a thorough transcriptome screening, a *strictosidine synthase-like* (*SSL*) gene was found to be independently transferred from Brassicaceae to the root parasitic plant *Orobanche aegyptiaca* and to the shoot parasitic plant *Cuscuta australis*. Expression analyses also suggested that foreign *SSL* genes may retain certain functions in the recipient species^25^. C_4_ photosynthesis, a complex trait that confers higher productivity under warm and arid conditions, evolved independently many times from C_3_ ancestors. Christin and his colleagues^26^ revealed that the grass lineage *Alloteropsis* acquired two essential genes of the C_4_ pathway *via* a minimum of four independent lateral gene transfers from C_4_ taxa in the past 10 million years.

One of the most momentous events in the evolution of green plants is the colonization of land, which contributed tremendously to the establishment of the modern terrestrial environment. During their transition from aquatic to terrestrial environments, plants evolved some complex regulatory systems, body plans and other phenotypic novelties to conquer tremendous challenges, such as increased UV irradiation, drought, heat and microbial infection^13^. Many of the genes that were horizontally acquired by early land plants are involved in many plant-specific activities, including vascular development, plant defense, nitrogen recycling and the biosynthesis of starch, polyamines, hormones and glutathione^27^,^28^,^29^,^30^, suggesting the importance of HGT in adaption during plant colonization of land. Land plants evolved from charophycean green algae *c*. 480–490 million years ago^31^. The *Tm-1L* gene is present in all of the lineages of land plants and *Klebsormidium*, which belongs to the charophyte algae that comprise streptophytes with land plants. However, this gene is absent from chlorophyte green algae. These results suggest that green plants acquired the *Tm-1L* gene before the split of land plants with charophytic algae. The phylogenetic relationship between *Tm-1L* homologs revealed that green plants gained this gene through HGT events. Surprisingly, at least two different HGT events, which had respectively contributed to the origin of two domains of the protein encoded by the *Tm-1L* gene, were included in the origin of this gene. The charophytic algae *Klebsormidium* species have primitive body plans, and most that have adapted to land also can survive in fresh water. In addition, some *Klebsormidium* species also show tolerance to typical terrestrial stresses, such as drought, freezing and high-intensity light^4^,^32^,^33^. The ubiquity of the *Tm-1L* gene in land plants and in the charophytic algae *Klebsormidium* suggests that the functions of this gene include a wide range of selectivity and might have been involved in the adaptive evolution from aquatic to land environments.

### Putative interaction between UPF0261- and TBST-domain-containing proteins

In cell biology, proteins rarely perform functions alone. Many molecular and biological processes within a cell are carried out by the synergy of a large number of protein components that are organized by their protein-protein interactions, which is a fundamental mechanism that underlies virtually all biological processes. Because many important interactions between proteins are conserved across lineages and/or species, the maintenance of these relationships would lead to a high degree of co-evolution between genetic matrices of proteins with interactive relationships^34^. A correlation between genetic matrices of two proteins can quantitatively measure the co-evolution between them and, therefore, make specific and impartial inferences regarding probable protein-protein interactions^35^. In this work, we noticed that if there is a TBST-domain-encoding gene in a bacterial, archaeal or fungal genome, the genome generally has at least one UPF0261-domain-encoding gene. In addition, most genomes containing these two genes only possess one copy of each. More importantly, both the methods of Mirrortree and distance revealed that these two genes co-evolved in bacteria, archaea and fungi. Although our results do not provide structural evidence of the direct binding between the UPF0261- and TBST-domain-containing proteins, high evolutionary correlation and putative interaction between them were suggested. When the genome acquired one of the UPF0261- and TBST-domain-encoding genes through HGT, the interaction between them may have forced the plant genome to gain the other.

### Evolutionary scenarios

Two independent HGT events contributed to the acquisition of the UPF0261 and TBST domains in the *Tm-1L* gene of the land plant ancestor, respectively. However, unlike the genes in other species, the plant *Tm-1L* gene encodes a protein with both of these domains. Thus, a gene fusion event is suggested between the genes encoding UPF0261- and TBST-domain-containing proteins in the genome of the land plant ancestor. The story of the evolution of land plant *Tm-1L* genes is dramatic and fascinating, and at least two steps were included in the origin of this functionally important gene (Fig. 4B). In the first step, the ancestor of streptophytes acquired two bacterial genes through two independent HGT events. These two genes encode UPF0261- and TBST-domain-containing proteins, respectively, and are located in adjacent regions in the plant genome. In the second step, the ancestor of streptophytes evolved the *Tm-1L* gene through the fusion of the UPF0261-domain-encoding gene with the TBST-domain-encoding gene. In our investigation, the origin of the plant *Tm-1L* gene is the result of a combination of two independent HGT events and one gene fusion event.

Both HGT and gene fusion are of evolutionary importance in creating new genes with novel functions^36^. It has been suggested that the benefit and the driving force behind gene fusion is to lower the regulational load of multiple interacting gene products^37^. The accretion of multiple domains appears to be one of the important routes for increasing functional complexity in the evolution of multicellular eukaryotes^38^. In addition, gene fusion is suggested to be one of the main principles of functional design in signal transduction systems, such as sugar phosphotransferase systems and receptor kinases^39^,^40^. The fusion of UPF0261 and TBST domains led to the origin of the Tm-1L protein in plants. The origin of the plant Tm-1L protein may also be included the functional design in signal transduction systems because the C-terminal domain TBST has been suggested to possess a potential role in signal recognition/receiving and signal transduction.

The combination of HGT and gene fusion has been previously reported, and two types of mechanisms have been suggested. One mechanism is the HGT of fused genes. For example, the origin of the fern neochrome involved the retrotransposition of a phototropin gene, fusion with a phytochrome and HGT from hornworts to ferns^41^. The other mechanism is the fusion of the horizontally acquired gene with a pre-existing gene in the acceptor genome. Phylogenetic evidence has revealed that plant expansin genes that encode cell-wall-loosening proteins were transferred from plants to bacteria. Subsequently, expansin genes have been suggested to be independently fused to the genes that code for an endoglucanase domain or for a carbohydrate-binding module in bacteria^42^. Here, we report a rare case of the origin of plant genes through the combination of HGT and gene fusion. In this case, the ancestral genome of land plants independently acquired two genes with a putatively functional interaction through two HGT events, and a subsequent gene fusion event that joined these two genes led to the origin of the *Tm-1L* gene in plants.

## Materials and Methods

### Sequence data sources

To identify the genes encoding Tm-1L proteins in plants, the protein sequence of the tomato gene *SlTm-1* was used as a query to in the Phytozome^43^, NCBI *nr* protein and spruce genome project^44^ databases. If a protein sequence satisfied E ≤ 1E-10, it was selected as a candidate protein. Then, Pfam^45^ was used to predict the UPF0261 and TIM-barrel signal transduction (TBST) domains. The new *Tm-1L* sequences that were detected in plants were used reiteratively to search the respective sequence database.

To identify the homologs of plant *Tm-1L* genes, BLAST searches against the non-redundant (nr) protein sequence, NCBI EST and available eukaryotic genome databases were performed using the plant Tm-1L protein sequences as queries. The obtained hits were further analyzed *via* a Pfam search to confirm the presence of the UPF0261 and/or TBST domains in the protein structure. Protein sequences were sampled for a further combined phylogenetic analysis from representative groups within each domain of life (bacteria, archaea and eukaryotes) based on the BLASTP results.

### Phylogenetic tree reconstruction

All of the selected representative protein sequences were aligned using Clustal X^46^. The gaps and ambiguously aligned sites were removed manually. Phylogenetic analyses were performed using a maximum likelihood (ML) approach with PhyML version 3.0^47^ and a neighbor-joining (NJ) method using MEGA version 6.0^48^. The model of protein evolution that best fit the protein alignment, the proportion of invariable sites and the alpha parameter of the gamma distribution were estimated using the ModelGenerator program^49^. A total of 100 non-parametric bootstrap samplings were carried out to estimate the support level for each internal branch for both the ML and NJ trees. Phylogenetic trees were visualized using the explorer program in MEGA.

### Evaluation of genetic distance and co-evolution analyses

The Mirrortree web server^14^ was used to estimate the degree of co-evolution between TBST and UPF0261 proteins. In this analysis, the sequences of the UPF0261 and TBST domain regions in the *Arabidopsis* Tm-1L protein (AT5G66420) were used as two references.

All of the bacterial, archaeal and fungal genes encoding TBST-domain-containing proteins in the NCBI CDD database were retrieved. In this analysis, only the genes with RefSeq genomes were selected. Blast tools were further used to verify the existence of the genes encoding UPF0261-domain-containing proteins in these genomes. To test the putative co-evolution, the linear correlation coefficient between the genetic distance matrices of TBST and UPF0261 proteins was determined^50^. The TBST and UPF0261 protein sequences in these three lineages were aligned using Clustal X^46^. Distance matrices were computed for each of the alignments by MEGA using the Jones-Taylor-Thornton (JTT) model for amino acid replacement per site, which was also estimated by using the ModelGenerator program. For TBST and UPF0261 proteins within *n* genomes in common in their multiple sequence alignment, the correlation coefficient *r* could be calculated as follows:


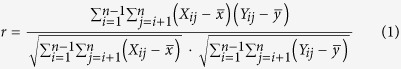


where 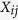 is the genetic distance for the TBST protein between genomes *i* and *j*, while 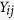 is the genetic distance for protein UPF0261 between genomes *i* and *j*.

## Additional Information

**How to cite this article**: Yang, Z. *et al*. Origin of the plant *Tm-1-like* gene *via* two independent horizontal transfer events and one gene fusion event. *Sci. Rep.* **6**, 33691; doi: 10.1038/srep33691 (2016).

## Supplementary Material

Supplementary Information

## Figures and Tables

**Table 1:** Pearson correlation coefficients for the co-evolution of the UPF0261 and TBST proteins using the RefSeq protein dataset and Mirrortree method.

Lineage	RefSeq proteins			Mirrortree		
	Genomes	*r*	*P*	Genomes	*r*	*P*
Common	416	0.7192	<0.0001	159	0.886	<0.0001
Bacteria	333	0.7735	<0.0001	136	0.906	<0.0001
Archaea	22	0.7185	<0.0001	3	0.990	<0.05
Fungi	61	0.8067	<0.0001	19	0.867	<0.0001
