# Supplementary material for: Origin of the plant Tm-1-like gene via two independent horizontal transfer events and one gene fusion event
Source: Sci Rep. 2016 Sep 20;6:33691. doi: 10.1038/srep33691 (PMC5028733; doi:10.1038/srep33691)
Supplement: Supplementary Information [file srep33691-s1.pdf]

# Supplementary Information

# Origin of the plant *Tm-1-like* gene *via* two independent horizontal transfer events and one gene fusion event

Zefeng Yang[1], Li Liu[1], Huimin Fang[1], Pengcheng Li[1], Shuhui Xu[1], Wei Cao[1], Chenwu Xu[1,#], Jinling Huang[2,#] , Yong Zhou[1,#]

1 Jiangsu Key Laboratory of Crop Genetics and Physiology/Co-Innovation Center for Modern Production Technology of Grain Crops, Key Laboratory of Plant Functional Genomics of the Ministry of Education, Yangzhou University, Yangzhou, 225009, China

2 Department of Biology, East Carolina University, Greenville, NC, 27858, USA

# Corresponding author: Chenwu Xu

Email: qtls@yzu.edu.cn

Jinling Huang

Email: huangj@ecu.edu

Yong Zhou

Email: zhouyong@yzu.edu.cn

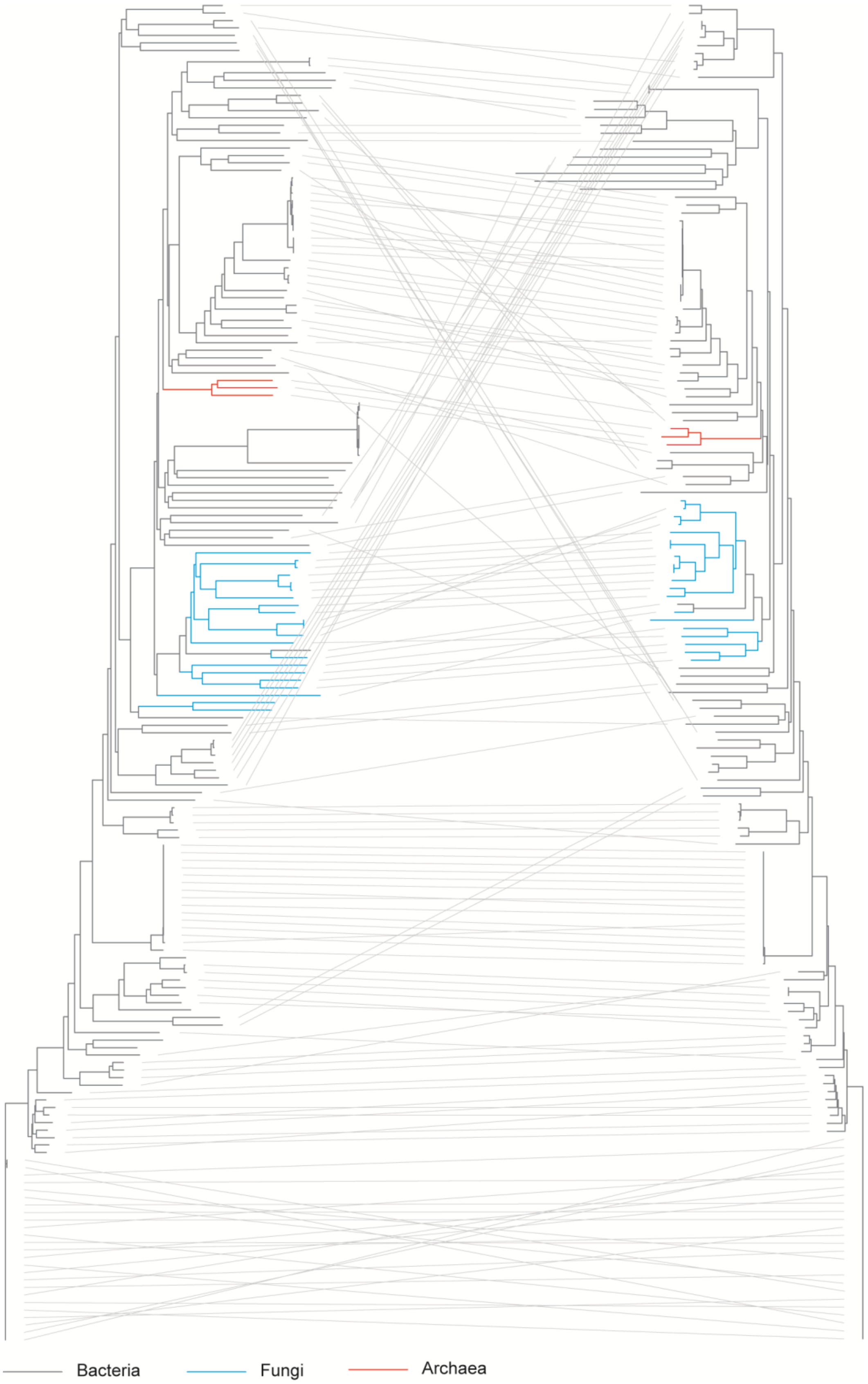


**Supplementary Figure S1** UPF0261 and TBST phylogenies of bacteria, archaea and fungi.

**Supplementary Table S1** List of *Tm-1L* genes in 18 representative streptophyte plant genomes

| Lineage | Species | Gene | Locus | Length | Intron | Chr/scaffold | Location |
|---|---|---|---|---|---|---|---|
| Charophyta | *Klebsormidium flaccidum* | *KfTm-1L* | KFL_000010590 | 745 | 11 | DF236950 | 379034..385338 |
| Bryophyta | *Physcomitrella patens* | *PpTm-1L* | Phpat.020G033800 | 742 | 9 | Chr20 | 4976232..4982023 |
| Lycophyte | *Selaginella moellendorffii* | *SmTm-1L* | 402546 | 741 | 8 | scaffold_0 | 3515380..3518137 |
| Gymnosperm | *Picea abies* | *PaTm-1L* | MA_38249g0010, MA_532686g0010 | 756 | 8 | MA_38249, MA_532686 | / |
| Basal angiosperm | *Amborella trichopoda* | *AmTm-1L* | LOC18422510 | 753 | 8 | NW_006494910 | c2909069-2865124 |
| | *Arabidopsis thaliana* | *AtTm-1L* | AT5G66420 | 754 | 8 | Chr5 | 26521674..26525081 |
| | *Vitis vinifera* | *VvTm-1L* | GSVIVG01018892001 | 756 | 8 | chr4 | 18877449..18887691 |
| | *Medicago truncatula* | *MtTm-1L* | Medtr8g076950 | 753 | 7 | chr8 | 32654902..32660930 |
| | *Eucalyptus grandis* | *EgTm-1L* | Eucgr.I02404 | 752 | 8 | scaffold_9 | 34690554..34697372 |
| Dicots | *Citrus sinensis* | *CsTm-1L* | orange1.1g004491m | 749 | 8 | scaffold00017 | 132259..136518 |
| | *Aquilegia coerulea* | *AcTm-1La* | Aquca_007_00265 | 755 | 8 | scaffold_7 | 2333433..2344601 |
| | *Aquilegia coerulea* | *AcTm-1Lb* | Aquca_007_00452 | 755 | 8 | scaffold_7 | 3740338..3751318 |
| | *Mimulus guttatus* | *MgTm-1L* | Migut.H02523 | 757 | 7 | scaffold_8 | 24733948..24737497 |
| | *Solanum lycopersicum* | *SlTm-1* | Solyc02g062560.2 | 754 | 8 | SL2.40ch02 | 28856001..28864037 |

| | | | | | | | |
|---|---|---|---|---|---|---|---|
| Monocots | *Oryza sativa* | *OsTm-1L* | LOC_Os06g27800 | 743 | 8 | Chr6 | 15738385..15744846 |
| | *Sorghum bicolor* | *SbTm-1L* | Sobic.010G120300 | 738 | 8 | Chr10 | 13695478..13701591 |
| | *Setaria italica* | *SiTm-1L* | Si005932m | 737 | 8 | scaffold_4 | 19160170..19165161 |
| | *Brachypodium distachyon* | *BdTm-1La* | Bradi2g26330 | 747 | 8 | Bd2 | 24754515..24761742 |
| | *Brachypodium distachyon* | *BdTm-1Lb* | Bradi1g38790 | 747 | 8 | Bd1 | 35398499..35405959 |
| | *Phoenix dactylifera* | *PdTm-1L* | LOC103703748 | 753 | 8 | NW_008246535 | 256851-268834 |

**Supplementary Table S2** List of 416 pairs of genes encoding UPF0261- and TBST-domain-containing proteins in bacteria, archaea and fungi

| Lineage | Species | ***UPF0261* Gene** | | ***TBST* Gene** | |
|---|---|---|---|---|---|
| | | **Locus** | **Location** | **Locus** | **Location** |
| Archaea | *Haladaptatus paucihalophilus* | WP_007980763.1 | NZ_AEMG01000014.1: complement(9617..10837) | WP_007980765.1 | NZ_AEMG01000014.1: complement(10839..11672) |
| Archaea | *Halalkalicoccus jeotgali* | WP_008414151.1 | NC_014298.1: (328077..329303, complement) | WP_008414152.1 | NC_014298.1: (329305..330138, complement) |
| Archaea | *Halapricum salinum* | WP_049993000.1 | NZ_BBMO01000001: complement(660447..661673 | WP_049993002.1 | NZ_BBMO01000001: complement(662100..662933) |
| Archaea | *Haloarcula amylolytica* | WP_008308240.1 | NZ_AOLW01000010: 230865..232091 | WP_008308236.1 | NZ_AOLW01000010: 229602..230435 |
| Archaea | *Haloarcula californiae* | WP_007190505.1 | NZ_AOLS01000106: 2586..3806 | WP_007190504.1 | NZ_AOLS01000106: 1325..2161 |
| Archaea | *Haloarcula hispanica* | WP_014030967.1 | NC_023010: complement(328935..330161) | WP_014030969.1 | NC_023010: complement(330582..331424) |
| Archaea | *Haloarcula japonica* | WP_004590970.1 | NZ_AOLY01000007: complement(47090..48298) | WP_004590972.1 | NZ_AOLY01000007: complement(48327..49163) |
| Archaea | *Haloarcula marismortui* | WP_011224960.1 | NC_006397: 152450..153670 | WP_049939228.1 | NC_006397: 151189..152025 |
| Archaea | *Haloarcula* sp. CBA1127 | WP_058991501.1 | NZ_BCNB01000001: complement(262875..264101) | WP_058991321.1 | NZ_BCNB01000001: complement(264531..265364) |
| Archaea | *Halobacteriaceae archaeon* SB9 | WP_058581343.1 | NZ_LOPU01000018: 386581..387807 | WP_058581341.1 | NZ_LOPU01000018: 385275..386114 |
| Archaea | *Halococcus agarilyticus* | WP_049903376.1 | NZ_BAFM01000021: 19050..20273 | WP_049903423.1 | NZ_BAFM01000021: 20359..21186 |
| Archaea | *Haloferax* sp. SB29 | WP_058572005.1 | NZ_LOPV01000158: | WP_058572010.1 | NZ_LOPV01000158: |

| | | | | | |
|---|---|---|---|---|---|
| | | | complement(18856..20091) | | complement(20125..20958) |
| Archaea | *Halopiger xanaduensis* | WP_013875895.1 | NC_015658: 317940..319166 | WP_013875893.1 | NC_015658: 316670..317503 |
| Archaea | *Halorubrum lipolyticum* | WP_008006199.1 | NZ_AOJG01000028: 107194..108432 | WP_008006198.1 | NZ_AOJG01000028: 106361..107194 |
| Archaea | *Halorubrum* sp. AJ67 | WP_048076344.1 | NZ_CBVY010000002: complement(283367..284326) | WP_048076337.1 | NZ_CBVY010000002: 275883..276716 |
| Archaea | *Halorubrum* sp. T3 | WP_017342377.1 | NZ_JH815329: 200507..201730 | WP_017342376.1 | NZ_JH815329: 199674..200510 |
| Archaea | *Halostagnicola larsenii* | WP_049954650.1 | NZ_CP007057: complement(61847..63058) | WP_049954652.1 | NZ_CP007057: complement(63489..64328) |
| Archaea | *Haloterrigena salina* | WP_008892585.1 | NZ_AOIS01000006: 22498..23724 | WP_008892583.1 | NZ_AOIS01000006: 21228..22067 |
| Archaea | *Haloterrigena turkmenica* | WP_012945082.1 | NC_013744: 244512..245732 | WP_012945080.1 | NC_013744: 243239..244078 |
| Archaea | *Natronococcus jeotgali* | WP_008422835.1 | NZ_AOIA01000091: complement(47049..48281) | WP_008422836.1 | NZ_AOIA01000091: complement(48358..49191) |
| Archaea | *Natronococcus occultus* | WP_015322307.1 | NC_019974: complement(3041141..3042349) | WP_015322308.1 | NC_019974: complement(3042351..3043184) |
| Archaea | *Salinarchaeum* sp. Harcht-Bsk1 | WP_020447178.1 | NC_021313: complement(2548572..2549804) | WP_020447180.1 | NC_021313: complement(2550262..2551098) |
| Bacteria | *[Clostridium] purinilyticum* | WP_050355459.1 | NZ_LGSS01000008: complement(133165..134385) | WP_050355458.1 | NZ_LGSS01000008: complement(132319..133146) |
| Bacteria | *[Clostridium] ultunense* | WP_025640931.1 | NZ_AZSU01000003: 25783..27003 | WP_025640932.1 | NZ_AZSU01000003: 27019..27840 |
| Bacteria | *Acetobacter aceti* | WP_010666563.1 | NZ_KB902575: complement(2738095..2739327) | WP_042788220.1 | NZ_JEOA01000008: complement(134680..135510) |

| | | | | | |
|---|---|---|---|---|---|
| Bacteria | *Acetobacterium bakii* | WP_050739260.1 | NZ_LGYO01000010: complement(75..1289) | WP_050741055.1 | NZ_LGYO01000037: complement(13654..14481) |
| Bacteria | *Acetobacterium dehalogenans* | WP_026394826.1 | NZ_AXAC01000024: complement(15466..16674) | WP_026394825.1 | NZ_AXAC01000024: complement(14610..15437) |
| Bacteria | *Acetobacterium woodii* | WP_014356784.1 | NC_016894: complement(2836836..2838044) | WP_014356783.1 | NC_016894: complement(2835762..2836589) |
| Bacteria | *Acetonema longum* | WP_004092559.1 | NZ_AFGF01000017: 113615..114832 | WP_004092562.1 | NZ_AFGF01000017: 114858..115685 |
| Bacteria | *Acholeplasma equifetale* | WP_026399708.1 | NZ_JHXL01000002: 156328..157542 | WP_026399709.1 | NZ_JHXL01000002: 157563..158390 |
| Bacteria | *Achromobacter arsenitoxydans* | WP_008160625.1 | NZ_AGUF01000033: complement(38162..39373) | WP_008160622.1 | NZ_AGUF01000033: complement(37320..38150) |
| Bacteria | *Achromobacter insuavis* | WP_006396077.1 | NZ_GL982453: complement(6688402..6689613) | WP_006396076.1 | NZ_GL982453: complement(6687560..6688390) |
| Bacteria | *Achromobacter* sp. DH1f | WP_025135787.1 | NZ_AYMF01000006: 152899..154110 | WP_025135788.1 | NZ_AYMF01000006: 154122..154952 |
| Bacteria | *Achromobacter* sp. LC458 | WP_046804746.1 | NZ_LBHW01000012: complement(119021..120235) | WP_046804745.1 | NZ_LBHW01000012: complement(118148..118978) |
| Bacteria | *Achromobacter* sp. Root170 | WP_056324149.1 | NZ_LMHN01000015: 656281..657492 | WP_056324151.1 | NZ_LMHN01000015: 657504..658334 |
| Bacteria | *Achromobacter* sp. Root565 | WP_056562789.1 | NZ_LMGO01000001: complement(2253092..2254303) | WP_056562787.1 | NZ_LMGO01000001: complement(2252249..2253079) |
| Bacteria | *Achromobacter* sp. Root83 | WP_057282520.1 | NZ_LMIU01000001: 371183..372394 | WP_057282521.1 | NZ_LMIU01000001: 372406..373236 |
| Bacteria | *Achromobacter* sp. RTa | WP_043542239.1 | NZ_JPYO01000007: complement(88647..89858) | WP_043542238.1 | NZ_JPYO01000007: complement(87805..88635) |

| | | | | | |
|---|---|---|---|---|---|
| Bacteria | *Achromobacter spanius* | WP_050445146.1 | NZ_LGVG01000002: 123856..125076 | WP_050445147.1 | NZ_LGVG01000002: 125088..125918 |
| Bacteria | *Acidisphaera rubrifaciens* | WP_048860690.1 | NZ_BANB01000165: complement(15908..18109) | WP_048860689.1 | NZ_BANB01000165: complement(15072..15905) |
| Bacteria | *Actinomadura madurae* | WP_024934958.1 | NZ_AWOO02000017: 38177..39373 | WP_021598830.1 | NZ_AWOO02000017: 39370..40191 |
| Bacteria | *Actinomadura oligospora* | WP_026413235.1 | NZ_JADG01000010: complement(136557..137765) | WP_026413234.1 | NZ_JADG01000010: complement(135730..136560) |
| Bacteria | *Actinomadura rifamycini* | WP_034518040.1 | NZ_AULB01000015: 8167..9420 | WP_026402685.1 | NZ_AULB01000015: 9417..10232 |
| Bacteria | *Actinomycetospora chiangmaiensis* | WP_018330347.1 | NZ_KB903217: 157645..158829 | WP_018330348.1 | NZ_KB903217: 158826..159656 |
| Bacteria | *Actinopolyspora erythraea* | WP_043572596.1 | NZ_KN214175: complement(429484..430686) | WP_043572594.1 | NZ_KN214175: complement(428619..429428) |
| Bacteria | *Actinopolyspora halophila* | WP_017974240.1 | NZ_AQUI01000002: 1977510..1978709 | WP_026152270.1 | NZ_AQUI01000002: 1978739..1979584 |
| Bacteria | *Actinopolyspora iraqiensis* | WP_026453720.1 | NZ_KE387069: complement(33433..34629) | WP_026453719.1 | NZ_KE387069: complement(32589..33431) |
| Bacteria | *Actinopolyspora mortivallis* | WP_026449078.1 | NZ_KB913024: complement(2222242..2223441) | WP_026449077.1 | NZ_KB913024: complement(2221388..2222194) |
| Bacteria | *Agrobacterium tumefaciens* | WP_026449078.1 | NZ_KB913024: complement(2222242..2223441) | WP_026449077.1 | NZ_KB913024: complement(2221388..2222194) |
| Bacteria | *Ahrensia* sp. R2A130 | WP_009465074.1 | NZ_AEEB01000022: complement(16748..18934) | WP_009465073.1 | NZ_AEEB01000022: complement(15915..16745) |
| Bacteria | *Alicyclobacillus herbarius* | WP_026961414.1 | NZ_AUMH01000005: 50538..51791 | WP_026961415.1 | NZ_AUMH01000005: 51775..52602 |
| Bacteria | *Alkaliphilus oremlandii* | WP_012160057.1 | NC_009922: 2362186..2363412 | WP_012160058.1 | NC_009922: 2363436..2364257 |

| | | | | | |
|---|---|---|---|---|---|
| Bacteria | *Allokutzneria albata* | WP_030429097.1 | NZ_JOEF01000005: complement(350819..352057) | WP_030429096.1 | NZ_JOEF01000005: complement(349995..350822) |
| Bacteria | *Aminiphilus circumscriptus* | WP_034264082.1 | NZ_JAFY01000001: complement(60161..61357) | WP_025745587.1 | NZ_JAFY01000001: complement(59264..60094) |
| Bacteria | *Amycolatopsis orientalis* | WP_043838740.1 | NZ_ASXH01000167: complement(158270..159514) | WP_043838739.1 | NZ_ASXH01000167: complement(157462..158283) |
| Bacteria | *Anaerobaculum hydrogeniformans* | WP_057940748.1 | NZ_ACJX03000001: complement(1097406..1098617) | WP_057940747.1 | NZ_ACJX03000001: complement(1096551..1097387) |
| Bacteria | *Anaerobaculum mobile* | WP_014806018.1 | NC_018024: complement(114293..115501) | WP_014806017.1 | NC_018024: complement(113437..114273) |
| Bacteria | *Ancylobacter* sp. FA202 | WP_018391584.1 | NZ_KB904819: complement(159554..160753) | WP_018391583.1 | NZ_KB904819: complement(158708..159538) |
| Bacteria | *Arhodomonas aquaeolei* | WP_018719490.1 | NZ_KB894821: 42003..43202 | WP_018719483.1 | NZ_KB894821: complement(35004..35831) |
| Bacteria | *Arsenophonus endosymbiont of Nilaparvata lugens* | WP_032116615.1 | NZ_KN173624: complement(8203..9420) | WP_032116614.1 | NZ_KN173624: complement(7357..8187) |
| Bacteria | *Arsenophonus nasoniae* | WP_026822523.1 | NZ_AUCC01000022: complement(30880..32097) | WP_026822522.1 | NZ_AUCC01000022: complement(30034..30864) |
| Bacteria | *Aurantimonas coralicida* | WP_024350891.1 | NZ_BBWN01000017: 85938..88103 | WP_029703199.1 | NZ_BBWN01000017: 88106..88954 |
| Bacteria | *Azospirillum halopraeferens* | WP_029010042.1 | NZ_AUCF01000033: 7951..9207 | WP_029010043.1 | NZ_AUCF01000033: 9209..10045 |
| Bacteria | *Bacillus farraginis* | WP_058004866.1 | NZ_LMCA01000015: 25045..26256 | WP_058004867.1 | NZ_LMCA01000015: 26272..27096 |
| Bacteria | *Bacillus massilioanorexius* | WP_019244219.1 | NZ_CAPG01000088: 10405..11637 | WP_026089424.1 | NZ_CAPG01000088: 11625..12455 |

| | | | | | |
|---|---|---|---|---|---|
| Bacteria | *Bacillus megaterium* | WP_026682232.1 | NZ_KI518608: complement(94781..96007) | WP_026682231.1 | NZ_KI518608: complement(93955..94779) |
| Bacteria | *Bacillus* sp. FJAT-18019 | WP_053491715.1 | NZ_LITP01000001: Unknown | WP_053491716.1 | NZ_LITP01000001: Unknown |
| Bacteria | *Bacillus* sp. UNC438CL73TsuS30 | WP_026572856.1 | NZ_AXVA01000011: 22971..24212 | WP_026572857.1 | NZ_AXVA01000011: 24199..25041 |
| Bacteria | *Bacillus thermoamylovorans* | WP_034767191.1 | NZ_CCRF01000009: 22069..23295 | WP_034767194.1 | NZ_CCRF01000009: 23296..24126 |
| Bacteria | bacteria symbiont BFo1 of *Frankliniella occidentalis* | WP_048917260.1 | / | WP_048917259.1 | / |
| Bacteria | bacterium mt3 | WP_054949746.1 | NZ_CTDZ01000009: complement(1382501..1383742) | WP_054949745.1 | NZ_CTDZ01000009: complement(1381659..1382477) |
| Bacteria | bacterium UASB270 | WP_045434693.1 | / | WP_045434691.1 | / |
| Bacteria | *Belnapia moabensis* | WP_043340154.1 | NZ_JQKB01000025: 72844..74061 | WP_043340156.1 | NZ_JQKB01000025: 74042..74872 |
| Bacteria | *Beutenbergia cavernae* | WP_015884276.1 | NC_012669: Unknown | WP_015884275.1 | NC_012669: Unknown |
| Bacteria | *Bilophila wadsworthia* | WP_005027753.1 | NZ_KE150238: 161393..162586 | WP_005027754.1 | NZ_KE150238: 162640..163467 |
| Bacteria | *Bordetella bronchiseptica* 99-R-0433 | WP_033465887.1 | NZ_JGWN01000100: complement(37424..38632) | WP_003810459.1 | NZ_JGWN01000100: complement(36584..37414) |
| *Bacteria* | *Bordetella parapertussis* | WP_010928219.1 | NZ_LRII01000001: complement(1106629..1107837) | WP_010928220.1 | NZ_LRII01000001: complement(1105789..1106619) |
| Bacteria | *Bordetella pertussis* strain I475 | WP_010930245.1 | NZ_CP010347: complement(1724001..1725209) | WP_010930246.1 | NZ_CP010347: complement(1723161..1723991) |
| Bacteria | *Bosea* sp. AAP35 | WP_054141202.1 | NZ_LJHQ01000003: complement(288970..291210) | WP_054141413.1 | NZ_LJHQ01000003: complement(288086..288961) |

| | | | | | |
|---|---|---|---|---|---|
| Bacteria | *Bosea* sp. Root483D1 | WP_057191436.1 | NZ_LMJW01000020: 265882..268116 | WP_057191437.1 | NZ_LMJW01000020: 268113..268988 |
| Bacteria | *Bradyrhizobium lablabi* | WP_057856136.1 | NZ_LLYB01000033: complement(9942..12179) | WP_057856135.1 | NZ_LLYB01000033: complement(9106..9939) |
| Bacteria | *Bradyrhizobium manausense* | WP_057758767.1 | NZ_LJYG01000112: 628115..630331 | WP_057758769.1 | NZ_LJYG01000112: 630335..631168 |
| Bacteria | *Bradyrhizobium oligotrophicum* | WP_015666016.1 | NC_020453: 3259529..3261745 | WP_042339458.1 | NC_020453: 3261751..3262596 |
| Bacteria | *Bradyrhizobium* sp. Ai1a-2 | WP_027578857.1 | NZ_AUEZ01000006: complement(124589..126805) | WP_027578856.1 | NZ_AUEZ01000006: complement(123752..124585) |
| Bacteria | *Bradyrhizobium* sp. BTAi1 | WP_012044823.1 | NC_009485: complement(5049586..5051805) | WP_012044822.1 | NC_009485: complement(5048735..5049580) |
| Bacteria | *Bradyrhizobium* sp. DOA9 | WP_025035422.1 | NZ_DF820425: complement(3956602..3958812) | WP_025035421.1 | NZ_DF820425: complement(3955765..3956598) |
| Bacteria | *Bradyrhizobium* sp. LTSP849 | WP_045010848.1 | NZ_JYMR01000012: 293154..295370 | WP_045010849.1 | NZ_JYMR01000012: 295374..296207 |
| Bacteria | *Bradyrhizobium* sp. LTSP857 | WP_045001001.1 | NZ_JYMS01000007: 765046..767262 | WP_045001003.1 | NZ_JYMS01000007: 767266..768099 |
| Bacteria | *Bradyrhizobium* sp. ORS 278 | WP_011927607.1 | NC_009445: complement(4975423..4977642) | WP_011927606.1 | NC_009445: complement(4974572..4975417) |
| Bacteria | *Bradyrhizobium* sp. ORS 285 | WP_006609916.1 | NZ_CAFH01000027: 70496..72715 | WP_006609917.1 | NZ_CAFH01000027: 72721..73566 |
| Bacteria | *Bradyrhizobium* sp. ORS 375 | WP_009028479.1 | NZ_CAFI01000186: complement(7416..9635) | WP_009028478.1 | NZ_CAFI01000186: complement(6566..7411) |
| Bacteria | *Bradyrhizobium* sp. S23321 | WP_015686994.1 | NC_017082: 4608432..4610648 | WP_015686995.1 | NC_017082: 4610652..4611485 |
| Bacteria | *Bradyrhizobium* sp. STM 3809 | WP_008965116.1 | NZ_CAFJ01000590: | WP_008965115.1 | NZ_CAFJ01000590: |

| | | | | | |
|---|---|---|---|---|---|
| | | | complement(20161..22380) | | complement(19310..20155) |
| Bacteria | *Bradyrhizobium* sp. Tv2a-2 | WP_024519131.1 | NZ_AXAI01000017: 39390..41606 | WP_024519132.1 | NZ_AXAI01000017: 41609..42442 |
| Bacteria | *Bradyrhizobium* sp. WSM2254 | WP_027548900.1 | NZ_KI421445: 205545..207761 | WP_027548901.1 | NZ_KI421445: 207765..208598 |
| Bacteria | *Bradyrhizobium* sp. WSM3983 | WP_027531282.1 | NZ_AXAY01000007: complement(238883..241099) | WP_027531281.1 | NZ_AXAY01000007: complement(238046..238879) |
| Bacteria | *Bradyrhizobium* sp. WSM471 | WP_007608672.1 | NZ_CM001442: complement(3517274..3519487) | WP_007608671.1 | NZ_CM001442: complement(3516437..3517270) |
| Bacteria | *Bradyrhizobium* sp. YR681 | WP_008139382.1 | NZ_AKIY01000238: complement(3741..5957) | WP_035966605.1 | NZ_AKIY01000238: complement(2904..3737) |
| Bacteria | *Bradyrhizobium valentinum* | WP_057855913.1 | NZ_LLXY01000090: 55138..57375 | WP_057855912.1 | NZ_LLXY01000090: 57378..58211 |
| Bacteria | *Brenneria goodwinii* | WP_048635954.1 | NZ_CGIG01000001: complement(501747..502958) | WP_048635953.1 | NZ_CGIG01000001: complement(500904..501734) |
| Bacteria | *Brevibacillus reuszeri* | WP_049741171.1 | NZ_LGIQ01000011: complement(39734..40981) | WP_049741170.1 | NZ_LGIQ01000011: complement(38914..39747) |
| Bacteria | *Budvicia aquatica* | WP_029096836.1 | NZ_KE386588: 166622..167833 | WP_029096837.1 | NZ_KE386588: 167843..168673 |
| Bacteria | *Burkholderia mallei* ATCC 23344 | WP_004206762.1 | NZ_LUFP01000085: 34025..35242 | WP_004201924.1 | NZ_LUFP01000085: 35245..36087 |
| Bacteria | *Burkholderia oklahomensis* | WP_010109433.1 | NZ_LOST01000093: complement(150558..151775) | WP_038801540.1 | NZ_LOST01000093: complement(149713..150555) |
| Bacteria | *Burkholderia pseudomallei* K96243 | WP_004546897.1 | NZ_CM003195: complement(2362803..2364020) | WP_004200988.1 | / |
| Bacteria | *Caldibacillus debilis* | WP_020156886.1 | NZ_KB912918: 33509..34747 | WP_026500106.1 | NZ_KB912918: 34731..35561 |
| Bacteria | *Candidatus Puniceispirillum* marinum | WP_013046653.1 | NC_014010: complement(1982938..1984158) | WP_013046651.1 | NC_014010: complement(1982090..1982923) |

| | | | | | |
|---|---|---|---|---|---|
| Bacteria | *Centipeda periodontii* | WP_006305316.1 | NZ_GL892076: complement(514734..515963) | WP_006305315.1 | NZ_GL892076: complement(513882..514712) |
| Bacteria | *Chelativorans* sp. BNC1 | WP_011583028.1 | NC_008254: complement(4012583..4013815) | WP_011583027.1 | NC_008254: complement(4011727..4012590) |
| Bacteria | *Clostridium argentinense* | WP_039633725.1 | NZ_AYSO01000017: 281060..282271 | WP_039633727.1 | NZ_AYSO01000017: 282288..283115 |
| Bacteria | *Clostridium botulinum* A str. ATCC 449 | WP_011949085.1 | NZ_LFOO01000006: 60850..62058 | WP_011949086.1 | NZ_LFOO01000006: 62083..62913 |
| Bacteria | *Clostridium drakei* | WP_032078519.1 | NZ_JIBU02000038: 6629..7837 | WP_032078520.1 | NZ_JIBU02000038: 7866..8696 |
| Bacteria | *Clostridium neonatale* | WP_058296486.1 | NZ_LN890328: complement(2624965..2626173) | WP_058296485.1 | NZ_LN890328: complement(2623989..2624819) |
| Bacteria | *Clostridium polynesiense* | WP_040211079.1 | NZ_CCXI01000040: 8333..9544 | WP_040211080.1 | NZ_CCXI01000040: 9655..10485 |
| Bacteria | *Clostridium scatologenes* | WP_029162168.1 | NZ_CP009933: 112528..113736 | WP_029162167.1 | NZ_CP009933: 113766..114596 |
| Bacteria | *Clostridium* sp. L74 | WP_053469563.1 | NZ_LITJ01000078: 278701..279909 | WP_053469564.1 | NZ_LITJ01000078: 279934..280764 |
| Bacteria | *Clostridium* sp. M62/1 | WP_008399240.1 | NZ_GG730317: 119406..120617 | WP_008399242.1 | NZ_GG730317: 120661..121488 |
| Bacteria | *Clostridium* sulfidigenes | WP_035135472.1 | NZ_JPMD01000050: complement(28487..29695) | WP_035135469.1 | NZ_JPMD01000050: complement(27617..28447) |
| Bacteria | *Coprothermobacter platensis* | WP_018963759.1 | NZ_KB899040: complement(1268378..1269610) | WP_018963758.1 | NZ_KB899040: complement(1267531..1268361) |
| Bacteria | *Coprothermobacter proteolyticus* | WP_012544577.1 | NC_011295: complement(1409623..1410855) | WP_012543487.1 | NC_011295: complement(1408778..1409608) |
| Bacteria | *Corynebacterium accolens* | WP_005281904.1 | NZ_GL397138: complement(833939..835156) | WP_005281903.1 | NZ_GL397138: complement(833076..833903) |

| | | | | | |
|---|---|---|---|---|---|
| Bacteria | *Corynebacterium* sp. KPL1818 | WP_023030825.1 | NZ_KI515760: complement(36227..37444) | WP_023030824.1 | NZ_KI515760: complement(35364..36191) |
| Bacteria | *Cryptosporangium arvum* | WP_035859346.1 | NZ_KK073874: complement(9085595..9086818) | WP_035859344.1 | NZ_KK073874: complement(9084774..9085598) |
| Bacteria | *Dermabacter hominis* | WP_034371397.1 | NZ_KN323183: complement(773059..774300) | WP_034371394.1 | NZ_KN323183: complement(772230..773057) |
| Bacteria | *Dermabacter* sp. HFH0086 | WP_016663580.1 | NZ_KE340308: complement(718147..719388) | WP_016663579.1 | NZ_KE340308: complement(717318..718145) |
| Bacteria | *Dethiosulfovibrio peptidovorans* | WP_005659904.1 | NZ_ABTR02000001: complement(872164..873360) | WP_050771353.1 | NZ_ABTR02000001: complement(871315..872100) |
| Bacteria | *Devosia* sp. LC5 | WP_035102221.1 | NZ_JNNO01000034: complement(201327..202523) | WP_035102218.1 | NZ_JNNO01000034: complement(200474..201316) |
| Bacteria | *Dickeya paradisiaca* | WP_015854705.1 | NZ_CM001857: complement(3496945..3498159) | WP_015854704.1 | NZ_CM001857: complement(3496104..3496934) |
| Bacteria | *Elioraea tepidiphila* | WP_019014842.1 | NZ_KB899922: complement(81354..82550) | WP_019014841.1 | NZ_KB899922: complement(80521..81351) |
| Bacteria | *Ensifer* sp. BR816 | WP_018240310.1 | NZ_KB905371: 109552..110760 | WP_018240311.1 | NZ_KB905371: 110738..111607 |
| Bacteria | *Ensifer* sp. USDA 6670 | WP_029963118.1 | NZ_ATWE01000018: complement(10479..11717) | WP_029963116.1 | NZ_ATWE01000018: complement(9668..10510) |
| Bacteria | *Enterobacter* sp. Bisph2 | WP_039077961.1 | NZ_JXAF01000025: 21962..23179 | WP_039077962.1 | NZ_JXAF01000025: 23196..24023 |
| Bacteria | *Enterobacteriaceae* bacterium B14 | WP_034864638.1 | NZ_DF158885: complement(127016..128227) | WP_034864637.1 | NZ_DF158885: complement(126172..127002) |
| Bacteria | *Enterococcus caccae* | WP_010771167.1 | NZ_KE136473: complement(217290..218519) | WP_010771166.1 | NZ_KE136473: complement(216212..217036) |
| Bacteria | *Enterococcus hirae* | WP_048720096.1 | NZ_JUPQ01000406: 1..937 | WP_048720099.1 | NZ_JUPQ01000406: 941..1771 |

| | | | | | |
|---|---|---|---|---|---|
| Bacteria | *Enterococcus* phoeniculicola | WP_010770070.1 | NZ_KB946329: complement(1312489..1313706) | WP_010770069.1 | NZ_KB946329: complement(1311645..1312472) |
| Bacteria | *Erwinia iniecta* | WP_052899396.1 | NZ_JRXE01000014: 4584..5804 | WP_052899397.1 | NZ_JRXE01000014: 5807..6637 |
| Bacteria | *Erwinia mallotivora* | WP_034940156.1 | NZ_JFHN01000065: complement(41458..42678) | WP_052018928.1 | NZ_JFHN01000065: complement(40628..41413) |
| Bacteria | *Erwinia oleae* | WP_034944728.1 | NZ_JNVB01000011: complement(70161..71387) | WP_034944725.1 | NZ_JNVB01000011: complement(69332..70159) |
| Bacteria | *Erwinia* sp. 9145 | WP_034916896.1 | NZ_JQNE01000001: complement(3156479..3157705) | WP_034916894.1 | NZ_JQNE01000001: complement(3155650..3156477) |
| Bacteria | *Erwinia toletana* | WP_017803477.1 | NZ_KB372811: 7819..9039 | WP_017803478.1 | NZ_KB372811: 9042..9872 |
| Bacteria | *Erwinia tracheiphila* | WP_016190305.1 | NZ_JXNU01000003: complement(1407339..1408559) | WP_016190304.1 | NZ_JXNU01000003: complement(1406509..1407336) |
| Bacteria | *Erwinia typographi* | WP_034892276.1 | NZ_JRUQ01000032: complement(45197..46417) | WP_034892274.1 | NZ_JRUQ01000032: complement(44367..45194) |
| Bacteria | *Ewingella americana* | WP_034795861.1 | NZ_JMPJ01000075: 70003..71217 | WP_034795864.1 | NZ_JMPJ01000075: 71230..72060 |
| Bacteria | *Flagellimonas* sp. DK169 | WP_055392226.1 | NZ_LCTZ01000002: 340068..341291 | WP_055392225.1 | NZ_LCTZ01000002: 339170..340006 |
| Bacteria | *Fodinicurvata fenggangensis* | WP_051609388.1 | NZ_JMLV01000011: complement(43471..45729) | WP_026988277.1 | NZ_JMLV01000011: complement(42651..43484) |
| Bacteria | *Fodinicurvata sediminis* | WP_022728895.1 | NZ_ATVH01000015: 312151..314406 | WP_022728896.1 | NZ_ATVH01000015: WP_022728896 |
| Bacteria | *Geminicoccus roseus* | WP_027133359.1 | NZ_KE386572: 1155278..1156468 | WP_027133360.1 | NZ_KE386572: 1156481..1157317 |
| Bacteria | *Gemmata obscuriglobus* | WP_010037484.1 | NZ_ABGO01000033: complement(34517..35791) | WP_033198058.1 | NZ_ABGO01000033: 36377..37204 |

| | | | | | |
|---|---|---|---|---|---|
| Bacteria | *Gemmata* sp. IIL30 | WP_052556044.1 | NZ_HG799465: 265004..265543 | WP_052556040.1 | NZ_HG799465: complement(263460..264287) |
| Bacteria | *Geobacillus subterraneus* | WP_033842841.1 | NZ_JQMN01000001: 496411..497649 | WP_033842843.1 | NZ_JQMN01000001: 497633..498463 |
| Bacteria | *Glaciibacter superstes* | WP_022885534.1 | NZ_ATWH01000008: 28285..29538 | WP_022885535.1 | NZ_ATWH01000008: 29546..30376 |
| Bacteria | *Gluconobacter morbifer* | WP_008851016.1 | NZ_AGQV01000001: 896534..897736 | WP_008851017.1 | NZ_AGQV01000001: 897739..898569 |
| Bacteria | *Halanaerobium hydrogeniformans* | WP_013405170.1 | NC_014654: 722275..723489 | WP_013405171.1 | NC_014654: 723506..724336 |
| Bacteria | *Halanaerobium praevalens* | WP_014553301.1 | NC_017455: complement(1249450..1250667) | WP_014553300.1 | NC_017455: complement(1248569..1249396) |
| Bacteria | *Halanaerobium saccharolyticum* | WP_005487951.1 | NZ_CAUI01000005: 719677..720888 | WP_005487953.1 | NZ_CAUI01000005: 720969..721802 |
| Bacteria | *Halomonas* sp. BC04 | WP_043517802.1 | NZ_AZQX01000356: complement(7714..8934) | WP_051496655.1 | NZ_AZQX01000356: complement(6884..7672) |
| Bacteria | *Halomonas* sp. KM-1 | WP_010626052.1 | NZ_BAEU01000020: complement(1466..2680) | WP_010626054.1 | NZ_BAEU01000020: complement(3185..4033) |
| Bacteria | *Hoeflea* sp. IMCC20628 | WP_047031005.1 | NZ_CP011479: complement(3338068..3339258) | WP_047031004.1 | NZ_CP011479: complement(3337208..3338056) |
| Bacteria | *Ideonella* sp. 201-F6 | WP_054021909.1 | NZ_BBYR01000065: complement(49124..50365) | WP_054021911.1 | NZ_BBYR01000065: complement(50939..51769) |
| Bacteria | *Ilumatobacter nonamiensis* | WP_040494102.1 | NZ_BAOL01000112: 48..1265 | WP_040494104.1 | NZ_BAOL01000112: 1262..2089 |
| Bacteria | *Jannaschia aquimarina* | WP_043918807.1 | NZ_JYFE01000037: complement(22029..24245) | WP_043918806.1 | NZ_JYFE01000037: WP_043918806 |
| Bacteria | *Jiangella muralis* | WP_053207659.1 | NZ_LFXL01000044: 38880..40121 | WP_053207660.1 | NZ_LFXL01000044: 40137..40967 |

| | | | | | |
|---|---|---|---|---|---|
| Bacteria | *Kitasatospora* sp. MY 5-36 | WP_049649984.1 | NZ_LFVW01000058: 11362..12597 | WP_049649985.1 | NZ_LFVW01000058: 12652..13476 |
| Bacteria | *Kluyvera intermedia* | WP_047368997.1 | NZ_CP011602: complement(122100..123317) | WP_047368996.1 | NZ_CP011602: complement(121256..122083) |
| Bacteria | *Komagataeibacter hansenii* | WP_003621924.1 | NZ_LUCI01000014: complement(22005..23357) | WP_003621921.1 | NZ_LUCI01000014: complement(21175..22005) |
| Bacteria | *Komagataeibacter intermedius* | WP_039735226.1 | NZ_BANF01000194: 12162..13394 | WP_039735227.1 | NZ_BANF01000194: 13372..14202 |
| Bacteria | *Komagataeibacter medellinensis* | WP_014106695.1 | NC_016027: complement(3116747..3117988) | WP_014106694.1 | NC_016027: complement(3115939..3116769) |
| Bacteria | *Komagataeibacter oboediens* | WP_010515931.1 | NZ_CADT01000040: 8854..10086 | WP_010515933.1 | NZ_CADT01000040: 10064..10894 |
| Bacteria | *Kutzneria albida* | WP_025360603.1 | NZ_KL570474: 7554..8771 | WP_025360604.1 | NZ_KL570474: 8768..9643 |
| Bacteria | *Lachnoanaerobaculum* sp. ICM7 | WP_009663593.1 | NZ_ALJL01000027: complement(19130..20347) | WP_009663606.1 | NZ_ALJL01000027: complement(18274..19104) |
| Bacteria | *Lachnoanaerobaculum* sp. OBRC5-5 | WP_007594527.1 | NZ_JH815185: complement(1950353..1951570) | WP_007594525.1 | NZ_JH815185: complement(1949498..1950328) |
| Bacteria | *Lactobacillus mellifer* | WP_046315202.1 | NZ_KQ034028: complement(90298..91512) | WP_046315200.1 | NZ_KQ034028: complement(89461..90291) |
| Bacteria | *Leminorella grimontii* | WP_027274915.1 | NZ_JMPN01000018: complement(31414..32628) | WP_027274916.1 | NZ_JMPN01000018: complement(30574..31401) |
| Bacteria | *Listeria aquatica* | WP_036072697.1 | NZ_AOCG01000009: complement(75417..76646) | WP_036072693.1 | NZ_AOCG01000009: complement(74591..75424) |
| Bacteria | *Lysinibacillus massiliensis* | WP_036179382.1 | NZ_JPVQ01000049: 116..1321 | WP_036179385.1 | NZ_JPVQ01000049: 1356..2186 |
| Bacteria | *Lysinibacillus xylanilyticus* | WP_049664865.1 | NZ_LFXJ01000005: 885230..886489 | WP_049664867.1 | NZ_LFXJ01000005: 886452..887276 |

| | | | | | |
|---|---|---|---|---|---|
| Bacteria | *Mesorhizobium alhagi* | WP_008833871.1 | NZ_AHAM01000017: complement(57..1253) | WP_008833870.1 | NZ_AHAM01000016: complement(11655..12479) |
| Bacteria | *Mesorhizobium australicum* | WP_015315629.1 | NC_019973: complement(1769405..1770601) | WP_015315628.1 | NC_019973: complement(1768538..1769395) |
| Bacteria | *Mesorhizobium erdmanii* | WP_027054358.1 | NZ_AXAE01000023: complement(79112..80308) | WP_027054357.1 | NZ_AXAE01000023: complement(78235..79101) |
| Bacteria | *Mesorhizobium huakuii* | WP_038649837.1 | NZ_CP006581: 4654363..4655559 | WP_038649841.1 | NZ_CP006581: 4655570..4656436 |
| Bacteria | *Mesorhizobium metallidurans* | WP_008874201.1 | NZ_CAUM01000060 : complement(38538..39734) | WP_008874200.1 | NZ_CAUM01000060 : complement(37659..38525) |
| Bacteria | *Mesorhizobium* sp. LC103 | WP_047146532.1 | NZ_LBCQ01000008 : 151827..154043 | WP_047146533.1 | NZ_LBCQ01000008 : 154048..154878 |
| Bacteria | *Mesorhizobium* sp. LNHC252B00 | WP_023760836.1 | NZ_AYWO01000017 : 112161..113357 | WP_023760837.1 | NZ_AYWO01000017 : 113368..114240 |
| Bacteria | *Mesorhizobium* sp. LSHC412B00 | WP_023725044.1 | NZ_AYWB01000001 : complement(455959..458118) | WP_023725043.1 | NZ_AYWB01000001 : complement(455119..455958) |
| Bacteria | *Mesorhizobium* sp. LSJC280B00 | WP_023675174.1 | NZ_AYVL01000004 : complement(40344..41540) | WP_023675173.1 | NZ_AYVL01000004 : complement(39460..40332) |
| Bacteria | *Mesorhizobium* sp. ORS3324 | WP_040973801.1 | NZ_CCMY01000176 : 18194..19390 | WP_040973802.1 | NZ_CCMY01000176 : 19403..20257 |
| Bacteria | *Mesorhizobium* sp. STM 4661 | WP_006328659.1 | NZ_CAAF010000026 : complement(60304..61500) | WP_006328658.1 | NZ_CAAF010000026 : complement(59428..60291) |
| Bacteria | *Mesorhizobium* sp. WSM3626 | WP_027144633.1 | NZ_AZUY01000025 : complement(32590..33786) | WP_027144632.1 | NZ_AZUY01000025 : complement(31723..32580) |
| Bacteria | *Methylobacterium variabile* | WP_048444391.1 | NZ_LABY01000073 : complement(25079..26284) | WP_048444390.1 | NZ_LABY01000073 : complement(24232..25074) |

| | | | | | |
|---|---|---|---|---|---|
| Bacteria | *Microbacterium azadirachtae* | WP_045272712.1 | NZ_JYIX01000037 ： complement(67805..69040) | WP_045272711.1 | NZ_JYIX01000037 ： complement(66963..67808) |
| Bacteria | *Microbacterium* sp. Root180 | WP_056122435.1 | NZ_LMHS01000003 ： complement(822474..823694) | WP_056122431.1 | NZ_LMHS01000003 ： complement(821525..822385) |
| Bacteria | *Microbispora rosea* | WP_030512351.1 | NZ_JNZQ01000054：23298..24533 | WP_030512352.1 | NZ_JNZQ01000054：24535..25365 |
| Bacteria | *Micromonospora carbonacea* | WP_043969468.1 | NZ_JXSX01000003 ： complement(1496740..1497963) | WP_043968370.1 | NZ_JXSX01000003 ： complement(1495914..1496738) |
| Bacteria | *Microvirga lupini* | WP_036363895.1 | NZ_KI912036：54060..55256 | WP_051455428.1 | NZ_KI912036：55253..56149 |
| Bacteria | *Microvirga* sp. BSC39 | WP_036345744.1 | NZ_JPUG01000007：4648..5865 | WP_036345740.1 | NZ_JPUG01000007：3342..4214 |
| Bacteria | *Mycobacterium mageritense* | WP_036435803.1 | NZ_CCBF010000001：4278269..4279522 | WP_036433727.1 | NZ_CCBF010000001：4276577..4277413 |
| Bacteria | *Mycobacterium* sp. Root135 | WP_056549661.1 | NZ_LMEZ01000001: complement(381242..382474) | WP_056549658.1 | NZ_LMEZ01000001: complement(380386..381168) |
| Bacteria | *Mycobacterium* sp. UM_WWY | WP_029369658.1 | NZ_AUWS01000024: complement(31963..33234) | WP_029369660.1 | NZ_AUWS01000024: complement(34073..34909) |
| Bacteria | *Natranaerobius thermophilus* | WP_012447345.1 | NC_010718: complement(929018..930235) | WP_012447344.1 | NC_010718: complement(928166..928993) |
| Bacteria | *Neorhizobium galegae* | WP_046635121.1 | NZ_CCRG01000053: 13374..14585 | WP_046635119.1 | NZ_CCRG01000053: 14582..15406 |
| Bacteria | *Nesiotobacter exalbescens* | WP_028482711.1 | NZ_KE383918: 443067..444269 | WP_028482712.1 | NZ_KE383918: 444288..445112 |
| Bacteria | *Nocardiopsis baichengensis* | WP_017559523.1 | NZ_ANAS01000038: complement(41000..42211) | WP_017559522.1 | NZ_ANAS01000038: complement(40165..40995) |
| Bacteria | *Nocardiopsis chromatogenes* | WP_017626150.1 | NZ_ANBH01000243: complement(7572..8664) | WP_017626149.1 | NZ_ANBH01000243: complement(6737..7567) |

| | | | | | |
|---|---|---|---|---|---|
| Bacteria | *Nocardiopsis gilva* | WP_017619322.1 | NZ_ANBG01000236: complement(1570..2823) | WP_017619321.1 | NZ_ANBG01000236: complement(736..1566) |
| Bacteria | *Nocardiopsis halophila* | WP_017538265.1 | NZ_ANAD01000067: 5981..7183 | WP_017538266.1 | NZ_ANAD01000067: 7188..8018 |
| Bacteria | *Nocardiopsis potens* | WP_017594438.1 | NZ_ANBB01000045: complement(50559..51815) | WP_017594437.1 | NZ_ANBB01000045: complement(49666..50496) |
| Bacteria | *Nonomuraea candida* | WP_043627653.1 | NZ_JOAG01000026: 149734..150972 | WP_043627655.1 | NZ_JOAG01000026: 150969..151784 |
| Bacteria | *Nonomuraea coxensis* | WP_020540311.1 | NZ_KB903940: 185115..186356 | WP_020540312.1 | NZ_KB903940: 186353..187180 |
| Bacteria | *Nonomuraea* sp. NBRC 110462 | WP_055501548.1 | NZ_BBZG01000001: 848394..849611 | WP_055501549.1 | NZ_BBZG01000001: 849608..850429 |
| Bacteria | *Nonomuraea* sp. SBT364 | WP_049570083.1 | NZ_LAVL01000100: complement(7616..8824) | WP_049570080.1 | NZ_LAVL01000100: complement(6798..7619) |
| Bacteria | *Noviherbaspirillum* sp. Root189 | WP_057293456.1 | NZ_LMHZ01000056: complement(287525..289774) | WP_057293368.1 | NZ_LMHZ01000056: complement(286686..287516) |
| Bacteria | *Oceanibaculum indicum* | WP_008942927.1 | NZ_AMRL01000001: complement(342390..343604) | WP_008942929.1 | NZ_AMRL01000001: complement(344083..344928) |
| Bacteria | *Oceanicola batsensis* | WP_009804053.1 | NZ_AAMO01000009: 85960..87165 | WP_040609010.1 | NZ_AAMO01000009: 87167..88030 |
| Bacteria | *Oceanicola granulosus* | WP_007257241.1 | NZ_CH724111: complement(127949..129172) | WP_007257242.1 | NZ_CH724111: complement(129184..130041) |
| Bacteria | *Paenibacillus borealis* | WP_042214031.1 | NZ_CP009285: 4322542..4323759 | WP_042214033.1 | NZ_CP009285: 4323785..4324615 |
| Bacteria | *Paenibacillus chondroitinus* | WP_047675029.1 | NZ_JUGY01000005: complement(200157..201386) | WP_047675028.1 | NZ_JUGY01000005: complement(199318..200148) |
| Bacteria | *Paenibacillus daejeonensis* | WP_020619288.1 | NZ_KB899667: 174483..175718 | WP_020619290.1 | NZ_KB899667: 176517..177347 |

| | | | | | |
|---|---|---|---|---|---|
| Bacteria | *Paenibacillus dendritiformis* | WP_006676043.1 | NZ_AHKH01000014: complement(35325..36557) | WP_006676042.1 | NZ_AHKH01000014: complement(34495..35328) |
| Bacteria | *Paenibacillus ehimensis* | WP_025844271.1 | NZ_JFHX01000001: 49850..51061 | WP_025844269.1 | NZ_JFHX01000001: complement(48781..49611) |
| Bacteria | *Paenibacillus elgii* | WP_010499345.1 | NZ_AFHW01000111: complement(21535..22746) | WP_010499347.1 | NZ_AFHW01000111: 22989..23819 |
| Bacteria | *Paenibacillus graminis* | WP_025703239.1 | NZ_CP009287: complement(3670351..3671568) | WP_025703240.1 | NZ_CP009287: complement(3669499..3670329) |
| Bacteria | *Paenibacillus odorifer* | WP_038573570.1 | NZ_CP009428: complement(6306579..6307787) | WP_038573568.1 | NZ_CP009428: complement(6305699..6306535) |
| Bacteria | *Paenibacillus pini* | WP_036647365.1 | NZ_BAZT01000004: complement(122858..124066) | WP_036647363.1 | NZ_BAZT01000004: complement(122012..122842) |
| Bacteria | *Paenibacillus* sp. A3 | WP_054973246.1 | NZ_JTHN01000059: 4803..6014 | WP_054973245.1 | NZ_JTHN01000059: complement(3727..4557) |
| Bacteria | *Paenibacillus* sp. A59 | WP_053779421.1 | NZ_LITU01000029: 118358..119605 | WP_053779422.1 | NZ_LITU01000029: 119598..120428 |
| Bacteria | *Paenibacillus* sp. AT5 | WP_055107250.1 | NZ_LN884299: 1023218..1024432 | WP_055107251.1 | NZ_LN884299: 1024475..1025299 |
| Bacteria | *Paenibacillus* sp. FJAT-22460 | WP_054404069.1 | NZ_LIUT01000003: complement(420648..421859) | WP_054404068.1 | NZ_LIUT01000003: complement(419768..420598) |
| Bacteria | *Paenibacillus* sp. FSL H7-0737 | WP_042192346.1 | NZ_CP009279: complement(6242955..6244163) | WP_042192344.1 | NZ_CP009279: complement(6242075..6242911) |
| Bacteria | *Paenibacillus* sp. FSL H8-237 | WP_036678525.1 | NZ_ASPV01000010: 41928..43136 | WP_036678527.1 | NZ_ASPV01000010: 43180..44016 |
| Bacteria | *Paenibacillus* sp. FSL P4-0081 | WP_042137505.1 | NZ_CP009280: 4327624..4328847 | WP_042137507.1 | NZ_CP009280: 4328867..4329697 |
| Bacteria | *Paenibacillus* sp. FSL R5-0345 | WP_042131317.1 | NZ_CP009281: | WP_042131316.1 | NZ_CP009281: |

| | | | | | |
|---|---|---|---|---|---|
| | | | complement(6259982..6261190) | | complement(6259104..6259940) |
| Bacteria | *Paenibacillus* sp. FSL R5-0912 | WP_042236594.1 | NZ_CP009282: 4046792..4048012 | WP_042236596.1 | NZ_CP009282: 4048032..4048862 |
| Bacteria | *Paenibacillus* sp. FSL R5-808 | WP_036642618.1 | NZ_ASPT01000035: complement(49713..50924) | WP_036642616.1 | NZ_ASPT01000035: complement(48824..49654) |
| Bacteria | *Paenibacillus* sp. FSL R7-269 | WP_036690714.1 | NZ_ASPS01000006: 45242..46459 | WP_036690716.1 | NZ_ASPS01000006: 46486..47316 |
| Bacteria | *Paenibacillus* sp. FSL R7-277 | WP_036727016.1 | NZ_ASPX01000058: complement(3996..5213) | WP_036727015.1 | NZ_ASPX01000058: complement(3140..3970) |
| Bacteria | Paenibacil*l*us sp. HGF5 | WP_036673086.1 | NZ_AEXS01000141: 23258..24469 | WP_009593972.1 | NZ_AEXS01000141: 24525..25355 |
| Bacteria | *Paenibacillus* sp. IHB B 3415 | WP_039292079.1 | NZ_JUEI01000002: complement(180813..182030) | WP_039292076.1 | NZ_JUEI01000002: complement(179959..180789) |
| Bacteria | *Paenibacillus* sp. IHBB 10380 | WP_044877313.1 | NZ_CP010976: 2515877..2517088 | WP_044877314.1 | NZ_CP010976: 2517096..2517929 |
| Bacteria | *Paenibacillus* sp. MSt1 | WP_036685340.1 | NZ_JNVM01000016: 21137..22348 | WP_036685948.1 | NZ_JNVM01000016: complement(20067..20900) |
| Bacteria | *Paenibacillus* sp. OSY-SE | WP_019423717.1 | NZ_ALKF01000199: complement(45049..46257) | WP_019423716.1 | NZ_ALKF01000199: complement(44195..45028) |
| Bacteria | *Paenibacillus* sp. P1XP2 | WP_036714087.1 | NZ_JRNV01000049: complement(29261..30487) | WP_036714086.1 | NZ_JRNV01000049: complement(28405..29229) |
| Bacteria | *Paenibacillus* sp. Soil787 | WP_056830177.1 | NZ_LMSP01000003: complement(174558..175787) | WP_056830174.1 | NZ_LMSP01000003: complement(173719..174549) |
| Bacteria | *Paenibacillus* sp. UNC217MF | WP_036666684.1 | NZ_JMLT01000008: complement(345441..346685) | WP_028531564.1 | NZ_JMLT01000008: complement(344608..345444) |
| Bacteria | *Paenibacillus taiwanensis* | WP_028544866.1 | NZ_KE384306: 139665..140873 | WP_028544867.1 | NZ_KE384306: 140951..141781 |
| Bacteria | *Paenibacillus vortex* | WP_006212407.1 | NZ_ADHJ01000048: complement(22462..23292) | WP_006212406.1 | NZ_ADHJ01000048: complement(22462..23292) |

| | | | | | |
|---|---|---|---|---|---|
| Bacteria | *Pantoea* sp. IMH | WP_024965815.1 | NZ_JFGT01000002: complement(1101641..1102858) | WP_024965814.1 | NZ_JFGT01000002: complement(1100811..1101638) |
| Bacteria | *Pantoea* sp. PSNIH2 | WP_038624200.1 | NZ_CP009866: 345055..346281 | WP_038624202.1 | NZ_CP009866: 346284..347114 |
| Bacteria | *Pedosphaera parvula* | WP_007412607.1 | NZ_ABOX02000001: complement(145151..146377) | WP_007418550.1 | NZ_ABOX02000069: 17678..18568 |
| Bacteria | *Pelobacter seleniigenes* | WP_029915664.1 | NZ_JOMG01000002: complement(2159813..2161009) | WP_029915662.1 | NZ_JOMG01000002: complement(2158941..2159771) |
| Bacteria | *Pelosinus fermentans* | WP_007958223.1 | NZ_CP010978: 3564263..3564523 | WP_007958222.1 | NZ_CP010978: complement(3563403..3564230) |
| Bacteria | *Peptoniphilus obesi* | WP_019131758.1 | NZ_HE978580: complement(166549..167766) | WP_019131757.1 | NZ_HE978580: complement(165690..166520) |
| Bacteria | *Photorhabdus heterorhabditis* | WP_054475940.1 | NZ_LJCS01000004: 62373..63593 | WP_054481442.1 | NZ_LJCS01000103: complement(6779..7609) |
| Bacteria | *Pirellula staleyi* | WP_012911825.1 | NC_013720: 3753976..3755217 | WP_012911826.1 | NC_013720: 3755335..3756174 |
| Bacteria | *Plantibacter* sp. Leaf314 | WP_056010795.1 | NZ_LMOB01000001: 2680455..2681759 | WP_056010798.1 | NZ_LMOB01000001: 2681762..2682586 |
| Bacteria | *Pleomorphomonas oryzae* | WP_026792166.1 | NZ_AUHB01000011: 38325..39539 | WP_026792167.1 | NZ_AUHB01000011: 39556..40389 |
| Bacteria | *Pontibacillus litoralis* | WP_036833306.1 | NZ_AVPG01000006: complement(65006..66244) | WP_036833304.1 | NZ_AVPG01000006: complement(64192..65022) |
| Bacteria | *Pragia fontium* | WP_047781684.1 | NZ_CP010423: 3072178..3073389 | WP_047781088.1 | NZ_CP010423: 2238410..2239237 |
| Bacteria | *Prauserella* sp. Am3 | WP_039505285.1 | NZ_JTJI01000015: 53965..55197 | WP_039505289.1 | NZ_JTJI01000015: 55199..56047 |
| Bacteria | *Propionibacterium acnes* | WP_023487410.1 | NZ_APCV01000094: 26451..27143 | WP_032504493.1 | NZ_AODA01000029: 56021..56869 |

| | | | | | |
|---|---|---|---|---|---|
| Bacteria | *Propionibacterium propionicum* | WP_014847830.1 | NC_018142: complement(3157174..3158394) | WP_014847829.1 | NC_018142: complement(3156332..3157162) |
| Bacteria | *Propionimicrobium lymphophilum* | WP_016455352.1 | NZ_KE150269: complement(536728..537954) | WP_016455351.1 | NZ_KE150269: complement(535877..536704) |
| Bacteria | *Propionispira raffinosivorans* | WP_019551997.1 | NZ_KB905839: 223309..224514 | WP_019551998.1 | NZ_KB905839: 224548..225378 |
| Bacteria | *Proteus hauseri* | WP_036911644.1 | NZ_AWXP01000001: 54706..55917 | WP_036911647.1 | NZ_AWXP01000001: 55934..56761 |
| Bacteria | Proteus vulgaris | WP_036932833.1 | NZ_KN150745: 231652..232863 | WP_036932835.1 | NZ_KN150745: 232880..233707 |
| Bacteria | *Providencia burhodogranariea* | WP_008910137.1 | NZ_KB233222: complement(96776..97999) | WP_008910136.1 | NZ_KB233222: complement(95938..96765) |
| Bacteria | *Providencia rustigianii* | WP_039855185.1 | NZ_GG703819: 546753..547970 | WP_006815178.1 | NZ_GG703819: 547985..548812 |
| Bacteria | *Providencia sneebia* | WP_008916532.1 | NZ_CM00177: 3319601..3320824 | WP_008916533.1 | NZ_CM00177: 3320835..3321662 |
| Bacteria | *Pseudonocardia asaccharolytica* | WP_037040531.1 | NZ_JNYD01000003: 54714..55898 | WP_051233149.1 | NZ_AUII01000013: complement(61007..61798) |
| Bacteria | *Pseudonocardia dioxanivorans* | WP_013676470.1 | NC_015312: complement(4725911..4727098) | WP_013676469.1 | NC_015312: complement(4725042..4725914) |
| Bacteria | *Pseudonocardia* sp. P1 | WP_010239421.1 | NZ_ADUJ01000708: 302..1504 | WP_010239424.1 | NZ_ADUJ01000708: 1501..2334 |
| Bacteria | *Pseudonocardia spinosispora* | WP_028936455.1 | NZ_KE386833: complement(1066908..1068137) | WP_028936454.1 | NZ_KE386833: complement(1066066..1066911) |
| Bacteria | *Pseudorhodoferax* sp. Leaf265 | WP_056659105.1 | NZ_LMMT01000004: 126988..129192 | WP_056659109.1 | NZ_LMMT01000004: 129170..130000 |
| Bacteria | *Pseudorhodoferax* sp. Leaf267 | WP_056179459.1 | NZ_LMMV01000004: 74307..76499 | WP_056179463.1 | NZ_LMMV01000004: 76483..77313 |
| Bacteria | *Pseudorhodoferax* sp. Leaf274 | WP_056897491.1 | NZ_LMNA01000001: | WP_056897492.1 | NZ_LMNA01000001: |

| | | | | | |
|---|---|---|---|---|---|
| | | | 455091..457304 | | 457282..458112 |
| Bacteria | *Rhizobacter* sp. Root404 | WP_056470898.1 | NZ_LMDS01000005: complement(264437..266662) | WP_056468103.1 | NZ_LMDS01000005: complement(263594..264424) |
| Bacteria | *Rhizobium gallicum* | WP_040114823.1 | NZ_CP006880: 467178..468374 | WP_040114824.1 | NZ_CP006880: 468387..469229 |
| Bacteria | *Rhizobium mongolense* | WP_022713863.1 | NZ_ATTQ01000003: 240416..241612 | WP_022713864.1 | NZ_ATTQ01000003: 241625..242467 |
| Bacteria | *Rhizobium* sp. Root1203 | WP_057470780.1 | NZ_LMCW01000029: complement(23240..24436) | WP_057470779.1 | NZ_LMCW01000029: complement(22386..23228) |
| Bacteria | *Rhizobium* sp. Root1220 | WP_056543371.1 | NZ_LMDG01000032: 395785..396978 | WP_056543374.1 | NZ_LMDG01000032: 396993..397835 |
| Bacteria | *Rhizobium sullae* | WP_027510035.1 | NZ_ATZB01000009: 25500..26696 | WP_027510036.1 | NZ_ATZB01000009: WP_027510036 |
| Bacteria | *Rhizobium vignae* | WP_037087774.1 | NZ_JNNU01000031: complement(13578..14774) | WP_037087771.1 | NZ_JNNU01000031: complement(12635..13468) |
| Bacteria | *Rhodococcus fascians* | WP_037144716.1 | NZ_JMEW01000021: 1017593..1018807 | WP_037144077.1 | NZ_JMEW01000021: 1018804..1019625 |
| Bacteria | *Rhodopirellula baltica* SH 1 | WP_011122999.1 | / | WP_011122998.1 | / |
| Bacteria | *Rhodospirillales bacterium* URHD0088 | WP_051473925.1 | NZ_JADL01000001: complement(530701..533037) | WP_027297941.1 | NZ_JADL01000001: complement(529818..530678) |
| Bacteria | *Rhodospirillum rubrum* | WP_011390591.1 | NC_017584: complement(3214359..3215579) | WP_011390590.1 | NC_017584: complement(3213516..3214346) |
| Bacteria | *Rhodovulum sulfidophilum* | WP_042461266.1 | NZ_CP015418: complement(2241184..2242380) | WP_042461264.1 | NZ_CP015418: complement(2240351..2241184) |
| Bacteria | *Roseiflexus castenholzii* | WP_012120903.1 | NC_009767: complement(3089277..3090512) | WP_012120902.1 | NC_009767: complement(3088442..3089275) |

| | | | | | |
|---|---|---|---|---|---|
| Bacteria | *Roseiflexus* sp. RS-1 | WP_011957489.1 | NC_009523: complement(3457268..3458494) | WP_011957488.1 | NC_009523: complement(3456432..3457265) |
| Bacteria | *Ruania albidiflava* | WP_022917014.1 | NZ_ATWL01000003: 75574..76797 | WP_022917015.1 | NZ_ATWL01000003: 76849..77679 |
| Bacteria | *Rubrobacter radiotolerans* | WP_041338934.1 | NZ_CP007515: 154825..156063 | WP_041338729.1 | NZ_CP007515: 156060..156884 |
| Bacteria | *Saccharomonospora cyanea* | WP_005456808.1 | NZ_CM001440: complement(2792542..2793750) | WP_005456807.1 | NZ_CM001440: complement(2791691..2792524) |
| Bacteria | *Saccharomonospora paurometabolica* | WP_028662672.1 | NZ_KI912259: complement(153816..154796) | WP_007023467.1 | NZ_KI912259: complement(152972..153814) |
| Bacteria | *Saccharomonospora saliphila* | WP_048876745.1 | NZ_KB912678: 77520..78525 | WP_019816331.1 | NZ_KB912678: 78527..79369 |
| Bacteria | *Saccharopolyspora erythraea* | WP_009948215.1 | NZ_AVCN01000257: complement(3883..5118) | WP_009948217.1 | NZ_AVCN01000257: complement(3044..3877) |
| Bacteria | *Saccharopolyspora rectivirgula* | WP_029719428.1 | NZ_JNVU01000025: 215856..217040 | WP_029719427.1 | NZ_JNVU01000025: 217058..217882 |
| Bacteria | *Saccharopolyspora spinosa* | WP_010306917.1 | NZ_GL877878: 691404..692618 | WP_010306920.1 | NZ_GL877878: 692615..693445 |
| Bacteria | *Salinibacterium* sp. PAMC 21357 | WP_010206263.1 | NZ_AHWA01000006: complement(439348..440595) | WP_010206262.1 | NZ_AHWA01000006: complement(438522..439355) |
| Bacteria | *Salinisphaera hydrothermalis* | WP_037339199.1 | NZ_APNK01000023: complement(27670..28878) | WP_037339196.1 | NZ_APNK01000023: WP_037339196 |
| Bacteria | *Salisaeta longa* | WP_022835817.1 | NZ_ATTH01000001: 1966588..1967799 | WP_022835818.1 | NZ_ATTH01000001: 1967925..1968755 |
| Bacteria | *Salsuginibacillus kocurii* | WP_051081212.1 | NZ_KB898633: 36131..37378 | WP_018924551.1 | NZ_KB898633: 37422..38249 |
| Bacteria | *Sciscionella* sp. SE31 | WP_031465618.1 | NZ_JALM01000015: 36834..38042 | WP_031465619.1 | NZ_JALM01000015: 38045..38887 |
| Bacteria | *Serratia liquefaciens* | WP_044548805.1 | NZ_DF820427: | WP_044548803.1 | NZ_DF820427: |

| | | | | | |
|---|---|---|---|---|---|
| | | | complement(357953..359170) | | complement(357108..357941) |
| Bacteria | *Shigella sonnei* | WP_049241669.1 | NZ_JVCS01000047: complement(29862..31079) | WP_049241667.1 | NZ_JVCS01000047: complement(29016..29849) |
| Bacteria | *Shimazuella kribbensis* | WP_028776227.1 | NZ_ATZF01000002: complement(447092..448306) | WP_028776226.1 | NZ_ATZF01000002: complement(446241..447083) |
| Bacteria | *Sinorhizobium arboris* | WP_027998635.1 | NZ_ATYB01000009: complement(64973..66169) | WP_027998634.1 | NZ_ATYB01000009: complement(64119..64961) |
| Bacteria | *Sinorhizobium meliloti* 1021 | WP_010967705.1 | NZ_ATYP01000023: 36786..37982 | WP_435974.1 | NZ_ATYP01000023: 37994..38836 |
| Bacteria | *Sodalis praecaptivus* | WP_025422359.1 | NZ_CP006569: 2509367..2510605 | WP_025422360.1 | NZ_CP006569: 2510595..2511425 |
| Bacteria | *Solirubrobacterales* bacterium URHD0059 | WP_028074450.1 | NZ_JIAT01000010: complement(21430..22635) | WP_028074449.1 | NZ_JIAT01000010: complement(20609..21433) |
| Bacteria | *Sporosarcina* sp. D27 | WP_025785394.1 | NZ_AZUC01000052: 1782..2993 | WP_025785395.1 | NZ_AZUC01000052: 3017..3847 |
| Bacteria | *Sporosarcina* sp. ZBG7A | WP_039043749.1 | NZ_JWIB01000034: complement(50288..51499) | WP_039043748.1 | NZ_JWIB01000034: complement(49434..50264) |
| Bacteria | *Streptomonospora alba* | WP_040275127.1 | NZ_JROO01000033: 29779..30999 | WP_040275128.1 | NZ_JROO01000033: 30996..31826 |
| Bacteria | *Streptomyces atriruber* | WP_055563890.1 | NZ_LIPN01000038: complement(7375..8628) | WP_055563863.1 | NZ_LIPN01000038: complement(6531..7352) |
| Bacteria | *Streptomyces catenulae* | WP_030278744.1 | NZ_JODY01000001: complement(274200..275438) | WP_030278741.1 | NZ_JODY01000001: complement(273373..274194) |
| Bacteria | *Streptomyces cattleya* | WP_014151446.1 | NC_017585: complement(735962..737608) | WP_014151449.1 | NC_017585: complement(733070..734050) |
| Bacteria | *Streptomyces celluloflavus* | WP_052856753.1 | NZ_JOEL01000013: complement(254091..255344) | WP_052856752.1 | NZ_JOEL01000013: complement(253197..254021) |
| Bacteria | *Streptomyces cellulosae* | WP_030661681.1 | NZ_JOEV01000002: | WP_030661664.1 | NZ_JOEV01000002: |

| | | | | | |
|---|---|---|---|---|---|
| | | | 128955..130217 | | complement(122092..122922) |
| Bacteria | *Streptomyces exfoliatus* | WP_024761330.1 | NZ_AZSS01000343: complement(3647..4897) | WP_024761329.1 | NZ_AZSS01000343: complement(2662..3504) |
| Bacteria | *Streptomyces kanamyceticus* | WP_055546257.1 | NZ_LIQU01000105: 1..964 | WP_055546259.1 | NZ_LIQU01000105: 992..1813 |
| Bacteria | *Streptomyces scabrisporus* | WP_020551151.1 | NZ_KB889662: complement(23898..25142) | WP_026218345.1 | NZ_KB889662: complement(23072..23896) |
| Bacteria | *Streptomyces showdoensis* | WP_046907641.1 | / | WP_046907640.1 | / |
| Bacteria | *Streptomyces* sp. CNB091 | WP_018958124.1 | NZ_KB898997: complement(49921..51174) | WP_018958123.1 | NZ_KB898997: complement(48996..49820) |
| Bacteria | *Streptomyces* sp. NRRL F-3213 | WP_037806101.1 | NZ_JOIQ01000001: 390378..391622 | WP_037806102.1 | NZ_JOIQ01000001: 391609..392430 |
| Bacteria | *Streptomyces* sp. NRRL F-5650 | WP_031034311.1 | NZ_JOGV01000001: complement(490909..492132) | WP_031034307.1 | NZ_JOGV01000001: complement(490000..490830) |
| Bacteria | *Streptomyces* sp. Root431 | WP_056642402.1 | NZ_LMEI01000006: 248852..250102 | WP_056642405.1 | NZ_LMEI01000006: 250231..251073 |
| Bacteria | *Streptomyces* sp. TAA204 | WP_028430312.1 | NZ_AUKW01000002: 598636..599868 | WP_028430313.1 | NZ_AUKW01000002: 599988..600815 |
| Bacteria | *Streptomyces* sp. TAA486 | WP_028437930.1 | NZ_AUEV01000021: complement(86434..87663) | WP_028437929.1 | NZ_AUEV01000021: complement(85617..86444) |
| Bacteria | *Streptomyces* sp. WMMB 322 | WP_055488033.1 | NZ_LIPX01000017: complement(73463..74692) | WP_055488032.1 | NZ_LIPX01000017: complement(72643..73473) |
| Bacteria | *Streptomyces* sp. WMMB 714 | WP_045863171.1 | NZ_LAND01000003: complement(82366..83604) | WP_045863170.1 | NZ_LAND01000003: complement(81461..82288) |
| Bacteria | *Streptomyces varsoviensis* | WP_030886468.1 | NZ_JOBF01000036: 48502..49770 | WP_030886472.1 | NZ_JOBF01000036: 50014..50835 |

| | | | | | |
|---|---|---|---|---|---|
| Bacteria | *Streptomyces venezuelae* | WP_055646478.1 | NZ_CP013129: complement(1273690..1274940) | WP_055644836.1 | NZ_CP013129: complement(1272756..1273598) |
| Bacteria | *Streptomyces vietnamensis* | WP_041127985.1 | NZ_CP010407: complement(1206059..1207333) | WP_041133610.1 | NZ_CP010407: complement(1205229..1206053) |
| Bacteria | *Succinispira mobilis* | WP_019879051.1 | NZ_KB913028: 687421..688638 | WP_019879052.1 | NZ_KB913028: 688656..689477 |
| Bacteria | *Terrisporobacter othiniensis* | WP_039679843.1 | NZ_JWHR01000093: 52091..53299 | WP_039679844.1 | NZ_JWHR01000093: 53323..54156 |
| Bacteria | *Tetrasphaera* sp. Soil756 | WP_055814716.1 | NZ_LMSE01000004: complement(27340..28551) | WP_055814711.1 | NZ_LMSE01000004: complement(26480..27343) |
| Bacteria | *Thermacetogenium phaeum* | WP_015049610.1 | NC_018870: complement(463426..464769) | WP_015049612.1 | NC_018870: complement(465615..466457) |
| Bacteria | *Thermanaerothrix daxensis* | WP_054520480.1 | NZ_LGKO01000002: complement(475055..476278) | WP_054520479.1 | NZ_LGKO01000002: complement(474198..475028) |
| Bacteria | *Thermoactinomyces daqus* | WP_033101852.1 | NZ_JPST01000036: complement(21324..21617) | WP_033101851.1 | NZ_JPST01000036: complement(20485..21309) |
| Bacteria | *Thermoactinomyces vulgaris* | WP_054095744.1 | NZ_LGKI01000145: complement(40887..42104) | WP_054095743.1 | NZ_LGKI01000145: complement(40041..40874) |
| Bacteria | *Thermoactinomycetaceae* bacterium GD1 | WP_044640401.1 | NZ_LN812102: complement(770346..771608) | WP_044640400.1 | NZ_LN812102: complement(769529..770368) |
| Bacteria | *Thermoanaerobacter ethanolicus* | WP_003869364.1 | NZ_AEYS01000004: complement(4642..5862) | WP_003869363.1 | NZ_AEYS01000004: complement(3791..4624) |
| Bacteria | *Thermomicrobium roseum* | WP_012642670.1 | NC_011961: complement(67225..68463) | WP_041437438.1 | NC_011961: complement(66360..67190) |
| Bacteria | *Thermorudis peleae* | WP_038037531.1 | NZ_JQMP01000001: 320900..322138 | WP_038037908.1 | NZ_JQMP01000001: 322185..323018 |

| | | | | | |
|---|---|---|---|---|---|
| Bacteria | *Thermovirga lienii* | WP_014163818.1 | NC_016148: complement(1754704..1755900) | WP_014163817.1 | NC_016148: complement(1753858..1754685) |
| Bacteria | *Timonella senegalensis* | WP_019147304.1 | NZ_HE978641: complement(765127..766347) | WP_019147303.1 | NZ_HE978641: complement(764235..765056) |
| Bacteria | *Tistrella mobilis* | WP_014744977.1 | NC_017956: 1629444..1630667 | WP_014744978.1 | NC_017956: 1630670..1631500 |
| Bacteria | *Variovorax* sp. URHB0020 | WP_051565477.1 | NZ_JHVQ01000005: 279983..282229 | WP_028249325.1 | NZ_JHVQ01000005: 282260..283090 |
| Bacteria | *Virgibacillus pantothenticus* | WP_050352496.1 | NZ_LGTO01000007: 1656547..1657773 | WP_050352497.1 | NZ_LGTO01000007: 1657775..1658599 |
| Bacteria | *Viridibacillus arenosi* | WP_038179451.1 | NZ_ASQA01000006: complement(79354..80562) | WP_038179449.1 | NZ_ASQA01000006: complement(78508..79338) |
| Bacteria | *Viridibacillus arvi* | WP_053418954.1 | NZ_LILB01000008: complement(472018..473226) | WP_053418953.1 | NZ_LILB01000008: complement(471172..472002) |
| Bacteria | *Wenxinia marina* | WP_018302558.1 | NZ_KN848374: complement(592747..593970) | WP_018302559.1 | NZ_KN848374: complement(591930..592760) |
| Bacteria | *Yersinia enterocolitica* subsp. enterocolitica 8081 | WP_005176149.1 | NZ_CGBA01000009: complement(78516..79748) | WP_011815345.1 | NZ_CTIY01000015: complement(45868..46698) |
| Bacteria | *Yersinia pekkanenii* | WP_049610150.1 | NZ_CWJL01000010: complement(77717..78979) | WP_049610148.1 | NZ_CWJL01000010: complement(76777..77607) |
| Bacteria | *Yersinia* pestis CO92 | WP_002211477.1 | NZ_CP009973: 3174814..3176031 | WP_002228178.1 | / |
| Bacteria | *Zavarzinella formosa* | WP_020468962.1 | NZ_JH636439: 1339596..1340801 | WP_020468960.1 | NZ_JH636439: complement(1338391..1339221) |
| Fungi | *Anthracocystis flocculosa* PF-1 | XP_007881871.1 | NW_006920892: complement(92421..93940) | XP_007881872.1 | NW_006920892: 94217..95279 |
| Fungi | *Arthrobotrys oligospora* ATCC | XP_011126097.1 | NW_011645730: | XP_011125999.1 | NW_011645727: |

| | | | | | |
|---|---|---|---|---|---|
| | 24927 | | complement(177821..179095) | | complement(215843..216831) |
| Fungi | *Aspergillus clavatus* NRRL 1 | XP_001269250.1 | NW_001517094: complement(2187963..2189297) | XP_001269251.1 | NW_001517094: 2189606..2190693 |
| Fungi | *Aspergillus flavus* NRRL3357 | XP_002380522.1 | NW_002477244: 1465953..1467236 | XP_002380525.1 | NW_002477244: 1471932..1472991 |
| Fungi | *Aspergillus fumigatus* Af293 | XP_752378.1 | NC_007194: 2608548..2609843 | XP_752377.1 | NC_007194: complement(2607310..2608266) |
| Fungi | *Aspergillus nidulans* FGSC A4 | XP_682200.1 | NT_107002: complement(214057..215385) | XP_682201.1 | NT_107002: 215546..216544 |
| Fungi | Aspergillus niger CBS 513.88 | XP_001392313.2 | NT_166524: complement(429893..431182) | XP_001392314.1 | NT_166524: 431829..432884 |
| Fungi | *Aspergillus nomius* NRRL 13137 | XP_015402016.1 | NW_015378550: complement(21405..22667) | XP_015403291.1 | NW_015378407: 1376..2414 |
| Fungi | *Aspergillus oryzae* RIB40 | XP_001825344.1 | NW_001884677: 1114606..1116069 | XP_001825346.1 | NW_001884677: 1121695..1122934 |
| Fungi | *Aureobasidium namibiae* CBS 147.97 | XP_013429585.1 | NW_013581205: complement(175483..176745) | XP_013429450.1 | NW_013581205: 176984..177826 |
| Fungi | *Aureobasidium subglaciale* EXF-2481 | XP_013348567.1 | NW_013566995: complement(109045..110307) | XP_013348569.1 | NW_013566995: 110547..111389 |
| Fungi | *Baudoinia panamericana* UAMH 10762 | XP_007671722.1 | NW_006911257: 415095..416378 | XP_007671721.1 | NW_006911257: complement(413864..414957) |
| Fungi | *Bipolaris maydis* ATCC 48331 | XP_014078654.1 | NW_014024926: complement(568701..569957) | XP_014078655.1 | NW_014024926: 570126..571175 |
| Fungi | *Bipolaris oryzae* ATCC 44560 | XP_007691418.1 | NW_006911432: 41121..42377 | XP_007691417.1 | NW_006911432: complement(39955..40878) |

| | | | | | |
|---|---|---|---|---|---|
| Fungi | *Bipolaris sorokiniana* ND90Pr | XP_007700716.1 | NW_006911902: 821525..823194 | XP_007700715.1 | NW_006911902: complement(820388..821417) |
| Fungi | *Bipolaris victoriae* FI3 | XP_014561279.1 | NW_014575386: 300830..302086 | XP_014561377.1 | NW_014575386: complement(299665..300586) |
| Fungi | *Bipolaris zeicola 2*6-R-13 | XP_007707408.1 | NW_006912055: 300859..302115 | XP_007707287.1 | NW_006912055: complement(299694..300615) |
| Fungi | *Blastomyces gilchristii* SLH14081 | XP_002623800.1 | NW_003101671: complement(1794848..1796128) | XP_002623801.1 | NW_003101671: 1796667..1798038 |
| Fungi | *Botrytis cinerea* B05.10 | XP_001555090.1 | NW_001814540: 328413..329684 | XP_001555089.1 | NW_001814540: complement(326808..327786) |
| Fungi | *Capronia coronata* CBS 617.96 | XP_007724410.1 | NW_006912896: 2173887..2175266 | XP_007724409.1 | NW_006912896: complement(2172634..2173473) |
| Fungi | *Capronia epimyces* CBS 606.96 | XP_007737709.1 | NW_006912912: complement(1223355..1224737) | XP_007737710.1 | NW_006912912: 1225332..1226171 |
| Fungi | *Cladophialophora carrionii* CBS 160.54 | XP_008725595.1 | NW_008481827: complement(2661190..2662572) | XP_008725596.1 | NW_008481827: 2663022..2663858 |
| Fungi | *Cladophialophora psammophila* CBS 110553 | XP_007741136.1 | NW_006912924: 416348..417730 | XP_007741135.1 | NW_006912924: complement(415021..415860) |
| Fungi | *Cladophialophora yegresii* CBS 114405 | XP_007759270.1 | NW_006913049: 966379..967767 | XP_007759269.1 | NW_006913049: complement(965110..965946) |
| Fungi | *Coniophora puteana* RWD-64-598 SS2 | XP_007764508.1 | NW_006913054: complement(757411..758807) | XP_007764509.1 | NW_006913054: 759171..760213 |
| Fungi | *Coniosporium apollinis* CBS 100218 | XP_007783822.1 | NW_006913307: complement(9995..11014) | XP_007783823.1 | NW_006913307: 11457..12504 |
| Fungi | *Cyphellophora europaea* CBS 101466 | XP_008720383.1 | NW_008481814: complement(120735..122165) | XP_008720387.1 | NW_008481814: complement(128362..129198) |

| | | | | | |
|---|---|---|---|---|---|
| Fungi | *Eutypa lata* UCREL1 | XP_007798609.1 | NW_006915772: 73881..75408 | XP_007798608.1 | NW_006915772: complement(72482..73522) |
| Fungi | *Exophiala aquamarina* CBS 119918 | XP_013262741.1 | NW_013550585: complement(4010862..4012256) | XP_013262143.1 | NW_013550585: 2224691..2225594 |
| Fungi | *Exophiala dermatitidis* NIH/UT8656 | XP_009160141.1 | NW_008751651: 2347189..2348568 | XP_009160140.1 | NW_008751651: complement(2345882..2346721) |
| Fungi | *Exophiala xenobiotica* | XP_013316699.1 | NW_013562492: complement(1620945..1622487) | XP_013316700.1 | NW_013562492: 1622706..1623548 |
| Fungi | *Fibroporia radiculosa* | XP_012181385.1 | NW_012133360: complement(6877..8166) | XP_012181386.1 | NW_012133360: 8411..9365 |
| Fungi | *Fonsecaea pedrosoi* CBS 271.37 | XP_013287225.1 | NW_013550614: 1963291..1964673 | XP_013287224.1 | NW_013550614: complement(1961970..1962809) |
| Fungi | *Histoplasma capsulatum* NAm1 | XP_001541274.1 | NW_001813980: 773150..776440 | XP_001541273.1 | NW_001813980: complement(771328..772300) |
| Fungi | *Leptosphaeria maculans* JN3 | XP_003839322.1 | NW_003533866: complement(2008817..2010076) | XP_003839323.1 | NW_003533866: 2010301..2011195 |
| Fungi | *Moesziomyces antarcticus* | XP_014656632.1 | NW_014639002: complement(662279..663667) | XP_014656633.1 | NW_014639002: 663956..665139 |
| Fungi | *Neofusicoccum parvum UCRNP2* | XP_007583355.1 | NW_006887908: 76852..78454 | XP_007583354.1 | NW_006887908: complement(75129..76269) |
| Fungi | *Neosartorya fischeri* NRRL 181 | XP_001264757.1 | NW_001509770: complement(2364537..2365829) | XP_001264758.1 | NW_001509770: 2366101..2367045 |
| Fungi | *Neurospora crassa* OR74A | XP_964779.1 | NC_026501: 6922584..6925468 | XP_964780.1 | NC_026501: complement(6921148..6922640) |
| Fungi | *Neurospora tetrasperma* FGSC 2508 | XP_009855735.1 | NW_009799179: complement(3038354..3039676) | XP_009855736.1 | NW_009799179: 3040133..3041143 |

| | | | | | |
|---|---|---|---|---|---|
| Fungi | *Parastagonospora nodorum* SN15 | XP_001799813.1 | NW_001884563: 180022..181453 | XP_001799812.1 | NW_001884563: complement(178966..179862) |
| Fungi | *Penicillium digitatum* Pd1 | XP_014532690.1 | NW_014574583: complement(5565624..5566922) | XP_014532692.1 | NW_014574583: 5569856..5570765 |
| Fungi | *Penicillium rubens* Wisconsin 54-1255 | XP_002560856.1 | NW_003020077: complement(1203709..1204989) | XP_002558911.1 | NW_003020074: 1141701..1142616 |
| Fungi | *Pestalotiopsis fici* W106-1 | XP_007826810.1 | NW_006917091: 124394..125761 | XP_007826809.1 | NW_006917091: complement(122808..123897) |
| Fungi | *Phaeoacremonium minimum* UCRPA7 | XP_007917479.1 | NW_006921397: complement(93161..94186) | XP_007917487.1 | NW_006921397: 94882..95794 |
| Fungi | *Podospora anserina* S mat+ | XP_001907064.1 | NW_001914853: complement(661552..662799) | XP_001907065.1 | NW_001914853: 663171..664090 |
| Fungi | *Pseudocercospora fijiensis* CIRAD86 | XP_007920164.1 | NW_006921532: complement(8583228..8584611) | XP_007919718.1 | NW_006921532: 8584871..8585965 |
| Fungi | *Pseudozyma hubeiensis* SY62 | XP_012186753.1 | NW_012133791: complement(296574..297962) | XP_012186754.1 | NW_012133791: 298220..299179 |
| Fungi | *Rasamsonia emersonii* CBS 393.64 | XP_013323502.1 | NW_013562625: 80206..81501 | XP_013323499.1 | NW_013562625: complement(74182..75168) |
| Fungi | *Rhinocladiella mackenziei* CBS 650.93 | XP_013268730.1 | NW_013550598: 1407726..1409105 | XP_013268729.1 | NW_013550598: complement(1406499..1407341) |
| Fungi | *Sclerotinia sclerotiorum* 1980 UF-70 | XP_001598468.1 | NW_001820835: complement(1456344..1457609) | XP_001598469.1 | NW_001820835: 1458476..1459690 |
| Fungi | *Serpula lacrymans* var. lacrymans S7.9 | XP_007322105.1 | NW_006763301: complement(734299..735683) | XP_007322106.1 | NW_006763301: 735792..736831 |
| Fungi | *Setosphaeria turcica* Et28A | XP_008029099.1 | NW_007360266:: complement(1366274..1367539) | XP_008029100.1 | NW_007360266: 1367727..1368671 |

| | | | | | |
|---|---|---|---|---|---|
| Fungi | *Sordaria macrospora* k-hell | XP_003352718.1 | NW_003546238: 1329903..1331204 | XP_003352717.1 | NW_003546238: complement(1328432..1329344) |
| Fungi | *Talaromyces marneffei* ATCC 18224 | XP_002151587.1 | NW_002196666: complement(1467301..1468705) | XP_002151588.1 | NW_002196666: 1468801..1470000 |
| Fungi | *Talaromyces stipitatus* ATCC 10500 | XP_002341451.1 | NW_002990113: 4070959..4072460 | XP_002341450.1 | NW_002990113: complement(4069374..4070629) |
| Fungi | *Trichoderma atroviride* IMI 206040 | XP_013942940.1 | NW_014013632: 110291..111774 | XP_013942939.1 | NW_014013632: complement(108958..109861) |
| Fungi | *Trichoderma reesei* QM6a | XP_006969368.1 | NW_006711178: complement(70077..71345) | XP_006969387.1 | NW_006711178: 71775..72771 |
| Fungi | *Trichoderma virens* Gv29-8 | XP_013961053.1 | NW_014013748: 342550..343971 | XP_013961052.1 | NW_014013748: complement(341198..342101) |
| Fungi | *Ustilago maydis* 521 | XP_011388427.1 | NC_026482: 37552..38943 | XP_011388426.1 | NC_026482: complement(36336..37313) |
| Fungi | *Zymoseptoria tritici* IPO323 | XP_003856186.1 | NC_018218: 2279893..2281296 | XP_003857466.1 | NC_018218: complement(2278392..2279562) |
